# HDAC7 promotes renal cancer progression by reprogramming branched-chain amino acid metabolism

**DOI:** 10.1126/sciadv.adt3552

**Published:** 2025-06-04

**Authors:** Hyeyoung Nam, Anirban Kundu, Suman Karki, Richard L. Kirkman, Darshan S. Chandrashekar, Jeremy B. Foote, Guofang Zhang, Wentao He, Sooryanarayana Varambally, Han-Fei Ding, Sunil Sudarshan

**Affiliations:** ^1^ Department of Urology, University of Alabama at Birmingham, Birmingham, AL, USA.; ^2^ Department of Urology and the University of Arizona Cancer Center, University of Arizona, Tucson, AZ, USA.; ^3^ Department of Pathology, University of Alabama at Birmingham, Birmingham, AL, USA.; ^4^ Department of Microbiology, University of Alabama at Birmingham, Birmingham, AL, USA.; ^5^ Sarah W. Stedman Nutrition and Metabolism Center and Duke Molecular Physiology Institute, Duke University, Durham, NC, USA.; ^6^ Department of Medicine, Division of Endocrinology, Metabolism and Nutrition, Duke University Medical Center, Durham, NC, USA.; ^7^ Birmingham Veterans Affairs Medical Center, Birmingham, AL, USA.

## Abstract

Clear cell renal cell carcinoma (ccRCC), the most common subtype of kidney cancer, exhibits notable metabolic reprogramming. We previously reported elevated HDAC7, a class II histone deacetylase, in ccRCC. Here, we demonstrate that HDAC7 promotes aggressive phenotypes and in vivo tumor progression in RCC. HDAC7 suppresses the expression of genes mediating branched-chain amino acid (BCAA) catabolism. Notably, lower expression of BCAA catabolism genes is strongly associated with worsened survival in ccRCC. Suppression of BCAA catabolism promotes expression of SNAIL1, a central mediator of aggressive phenotypes including migration and invasion. HDAC7-mediated suppression of the BCAA catabolic program promotes *SNAI1* messenger RNA transcription via NOTCH signaling activation. Collectively, our findings provide innovative insights into the role of metabolic remodeling in ccRCC tumor progression.

## Teaser

Epigenetic suppression of branched-chain amino acid catabolism by HDAC7 promotes renal tumor progression.

## INTRODUCTION

Malignant kidney tumors account for more than 14,000 patient deaths annually in the United States (*1*). Clear cell renal cell carcinoma (ccRCC), accounting for ~75% of cases, is the most common histologic subtype of renal cancer. Metastatic RCC remains largely incurable and is the primary cause of mortality. Despite substantial progress in early diagnosis, 30 to 40% of patients with ccRCC still present with metastases at initial diagnosis (*2*). While patients with localized RCC have a favorable prognosis, the 5-year survival rate for metastatic RCC is below 20% (*3*). Current therapeutic strategies have shown limited durable efficacy, underscoring the need for a better understanding of RCC metastasis to identify optimal therapeutic targets.

Cancer cells undergo metabolic reprogramming to meet their unique nutrient demands. This reprogramming is influenced by both environmental and intrinsic factors. These unique metabolic features of cancer cells have been exploited for developing therapeutic strategies and diagnostic tools. Emerging evidence indicates that additional metabolic alterations support aggressive behaviors driving metastasis. For example, cancer cells exploit alternative carbon sources to enhance migratory and invasive capabilities. Prior studies have demonstrated that certain nutrients, including pyruvate, amino acids, and lipids, facilitate the invasiveness and migratory abilities of cancer cells, as recently summarized (*4*).

Branched-chain amino acids (BCAAs; leucine, isoleucine, and valine) are essential amino acids acquired through diet that support protein synthesis. Alternatively, BCAAs can be catabolized in the mitochondria. Catabolism is initiated by BCAA transaminases (BCATs), which catalyze BCAA deamination to generate branched-chain keto acids (BCKAs). These BCKAs undergo irreversible decarboxylation by the branched-chain α-keto acid dehydrogenase (BCKDH) complex, the rate-limiting step in BCAA catabolism. Generated metabolites from this pathway include coenzyme A (CoA) derivatives, which can be used in processes such as the tricarboxylic acid (TCA) cycle. Notably, prior studies have shown that BCAA catabolic enzyme expression is down-regulated in various cancers including renal cancer (*5*). However, the mechanisms by which this occurs and their biological significance remain poorly understood. Here, we demonstrate a role for class II histone deacetylase 7 (HDAC7) in suppressing BCAA catabolism in RCC. This remodeling of BCAA metabolism leads to enhanced expression of snail family transcriptional repressor 1 (SNAIL1), which promotes aggressive phenotypes and tumor progression in RCC models. Collectively, our data indicate that inhibiting HDAC7 or its impact on BCAA metabolism could be targeted to mitigate the metastatic potential of RCC.

## RESULTS

### HDAC7 promotes invasive behavior and tumor progression in RCC

**Fig. 1.**
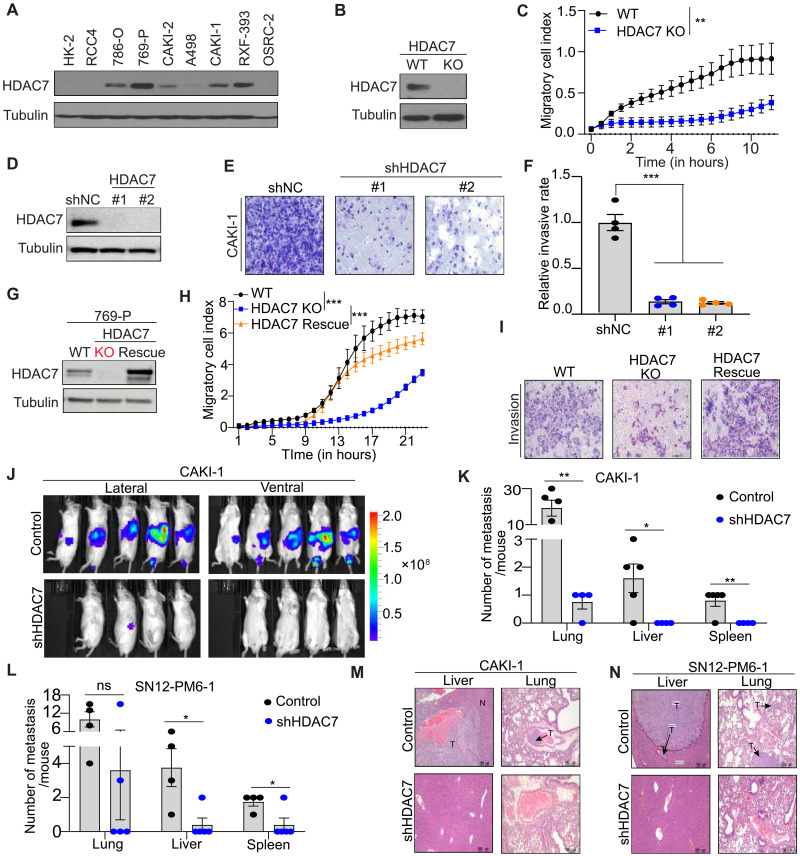
HDAC7 promotes invasive and migratory phenotypes in ccRCC. (**A**) Western blot analysis of HDAC7 expression in a panel of RCC cell lines relative to HK-2 renal epithelial cells. (**B**) Western blot analysis of HDAC7 in wild-type (WT) and HDAC7 CRISPR KO CAKI-1 cells. (**C**) Migration kinetics of RCC cells measured by the xCELLigence real-time cell analysis (RTCA) system (*n* = 4). (**D**) Western blot analysis of HDAC7 in CAKI-1 cells stably expressing negative control (NC) or shRNA for HDAC7. (**E** and **F**) Representative images and quantification of invasive cells from Boyden chamber invasion assay with Matrigel insert using CAKI-1 cells stably expressing NC or shRNA for HDAC7 (*n* = 4). (**G**) Western blot analysis of HDAC7 in 769-P cells expressing WT, HDAC7 KO, or HDAC7 rescue constructs. (**H**) Migration kinetics of RCC cells measured by the RTCA system (*n* = 4). (**I**) Representative images of Boyden chamber invasion assay with Matrigel inset using 769-P cells expressing WT, HDAC7 KO, or HDAC7 rescue constructs (*n* = 4). (**J**) Luciferase-expressing CAKI-1 cells were orthotopically implanted into the kidney capsule of SCID mice. Bioluminescence (BL) was used to monitor tumor metastasis at 6 weeks postinjection (*n* = 4 to 5). Lateral and ventral views provided. (**K** and **L**) Histologic quantification of spontaneous metastases to the lungs, liver, and spleen. (**M** and **N**) H&E staining of tissue sections from mice orthotopically implanted with control or shHDAC7-expressing RCC cells. Arrows indicate metastatic tumors (T) and normal tissues (N). In (C), (F), and (H), data are presented as means ± SEM and are representative of at least two independent experiments. Two-tailed Student’s *t* test was used for (C), (K), and (L), and ordinary one-way analysis of variance (ANOVA) with Tukey’s multiple comparisons test was used for (F) and (H). *P < 0.05, **P < 0.01, and ***P < 0.001; ns, not significant.

Our group recently demonstrated increased HDAC7 in ccRCC (*6*). Given these findings, we investigated the functional significance of increased HDAC7 in RCC. We examined HDAC7 expression in a panel of RCC cells compared to HK-2 renal epithelial cells. Multiple RCC cell lines, including 786-O, 769-P, CAKI-1, and RXF-393, displayed higher HDAC7 expression compared to HK-2 cells (Fig. 1A). We generated HDAC7 knockout (KO) CAKI-1 cells using the CRISPR-Cas9 system (Fig. 1B). HDAC7 KO cells exhibited decreased migratory and invasive behaviors (Fig. 1C and fig. S1A, respectively) without appreciable effects on doubling time (fig. S1B). We also generated HDAC7 knockdown CAKI-1 cells (Fig. 1D). Knockdown of HDAC7 using two independent short hairpin RNAs (shRNAs) led to reduced invasive phenotypes (Fig. 1, E and F). Similarly, HDAC7 KO in 769-P cells led to reduced migration/invasion, with these phenotypes rescued by reintroduction of *HDAC7* cDNA (Fig. 1, G to I, and fig. S1C). Furthermore, we evaluated HDAC7’s role in tumor biology using orthotopic RCC models with CAKI-1 and SN12-PM6-1 cells. Luciferase-expressing RCC cells transduced with shRNA control or shRNA targeting *HDAC7* were injected into the subcapsular kidney region of nonobese diabetic/severe combined immunodeficient (NOD/SCID) mice. HDAC7 ablation in both CAKI-1 and SN12-PM6-1 cells resulted in reduced overall tumor burden based on whole-body bioluminescent imaging (Fig. 1J and fig. S1D, respectively). In addition, orthotopic injection of RCC control cells developed histologically detectable metastases in multiple organs including the spleen, liver, and lung. In contrast, *HDAC7* knockdown in RCC cells led to reduced metastases in multiple organs based on histology (Fig. 1, K and L) and ex vivo bioluminescent imaging of tissues (fig. S1E). In the SN12-PM6-1 model, there was a trend toward reduced primary tumor growth with *HDAC7* knockdown; however, these data did not reach statistical significance (fig. S1E). Representative hematoxylin and eosin (H&E) staining demonstrating reduced liver and lung metastasis in HDAC7 knockdown cells is provided in Fig. 1 (M and N).

### HDAC7 suppresses the expression of genes encoding BCAA catabolic enzymes

**Fig. 2.**
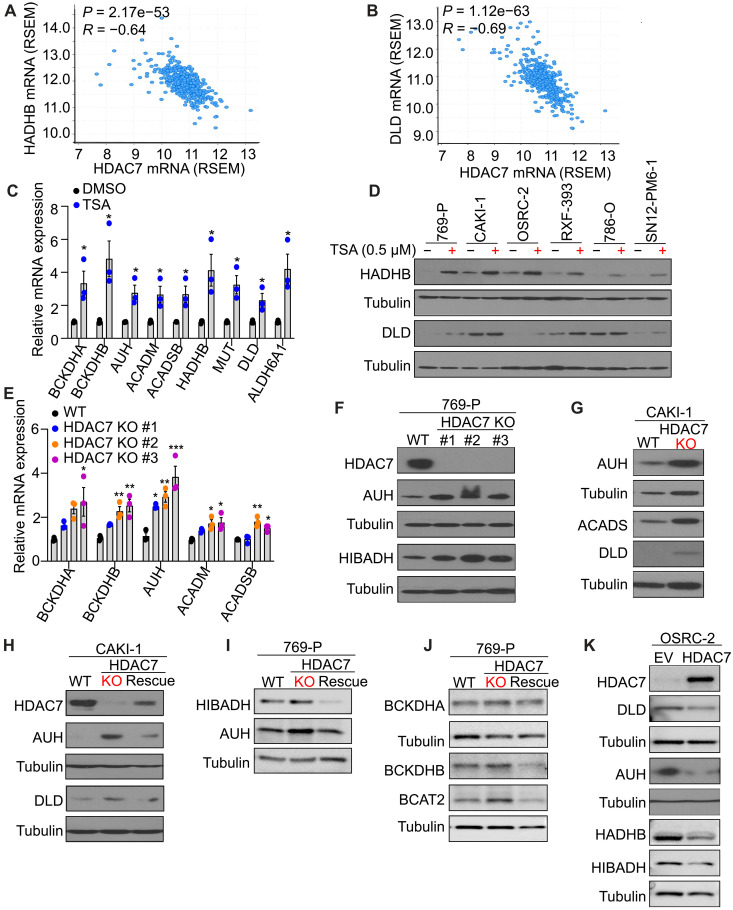
HDAC7 expression suppresses the expression of BCAA catabolic enzymes. (**A** and **B**) Correlation analysis between *HDAC7* and BCAA catabolic enzymes (*HADHB* and *DLD*) in the TCGA KIRC dataset for renal tumors. Data were extracted using the cBioPortal web server. RSEM, RNA-Seq by Expectation-Maximization. (**C**) Relative mRNA expression of BCAA catabolic enzymes in CAKI-1 cells treated with TSA for 24 hours (*n* = 3). DMSO, dimethyl sulfoxide. (**D**) Immunoblot analysis of indicated proteins in RCC cells treated with TSA for 24 hours. (**E**) Relative mRNA expression of BCAA catabolic enzymes in WT and three independent HDAC7 KO 769-P cell lines. (**F**) Western blot analysis of indicated proteins in WT and HDAC7 KO 769-P cells. (**G**) Immunoblot analysis of indicated proteins in WT and HDAC7 KO CAKI-1 cells. (**H**) Western blot analysis of indicated proteins in CAKI-1 cells expressing WT, HDAC7 KO, or HDAC7 rescue constructs. (**I** and **J**) Western blot analysis of indicated proteins in 769-P cells expressing WT, HDAC7 KO, or HDAC7 rescue constructs. (**K**) Western blot analysis of indicated proteins in OSRC-2 cells transiently expressing empty vector (EV) or HDAC7. In (C) and (E), data are presented as means ± SEM. Two-tailed Student’s *t* test was used for (C), and ordinary one-way ANOVA with Tukey’s multiple comparisons test was used for (E). *P < 0.05, **P < 0.01, and ***P < 0.001.

Our data demonstrating that HDAC7 promotes aggressive behaviors and tumor progression led us to examine the underlying mechanisms. Given that HDACs often repress gene expression, we performed correlation analysis of RNAs that negatively correlate with *HDAC7* mRNA in the The Cancer Genome Atlas (TCGA) dataset on ccRCC using GRACE (Genomic Regression Analysis of Coordinated Expression) as previously reported (*7*). KEGG (Kyoto Encyclopedia of Genes and Genomes) analysis revealed that the mRNA expression of BCAA catabolic enzymes inversely correlates with *HDAC7* (adjusted *P* = 4.88 × 10^−3^) (table S1). Multiple genes encoding BCAA catabolic enzymes exhibited statistically significant negative correlations with *HDAC7*, including *HADHB*, *DLD*, *HIBADH*, *MUT*, *ACADM*, *AUH*, and *ALDH6A1* (Fig. 2, A and B, and fig. S2, A to E). We initially assessed the effect of the pan-HDAC inhibitor trichostatin A (TSA) on the expression of BCAA catabolic enzymes. TSA treatment increased both mRNA and protein expression of BCAA catabolic enzymes (Fig. 2, C and D). We then compared the expression of BCAA catabolic enzymes in RCC cells with and without HDAC7. HDAC7 KO led to increased mRNA and protein expression of BCAA catabolic enzymes (Fig. 2, E to G, and fig. S3A). *HDAC7* reintroduction in KO cells diminished the increased expression of BCAA catabolic enzymes (Fig. 2, H and I). These effects were noted for core factors that mediate the initial catabolism of all three BCAAs including BCAT2, BCKDH alpha subunit (BCKDHA), and BCKDHB (Fig. 2J).

As shown in Fig. 1A, OSRC-2 RCC cells express low basal HDAC7. *HDAC7* cDNA delivery led to reduced protein levels of BCAA catabolic enzymes including AUH, DLD, HADHB, and HIBADH (Fig. 2K). Together, these data demonstrate that HDAC7 represses the expression of BCAA catabolic enzymes in RCC.

### BCAA catabolic enzyme expression is lost in renal cancer

**Fig. 3.**
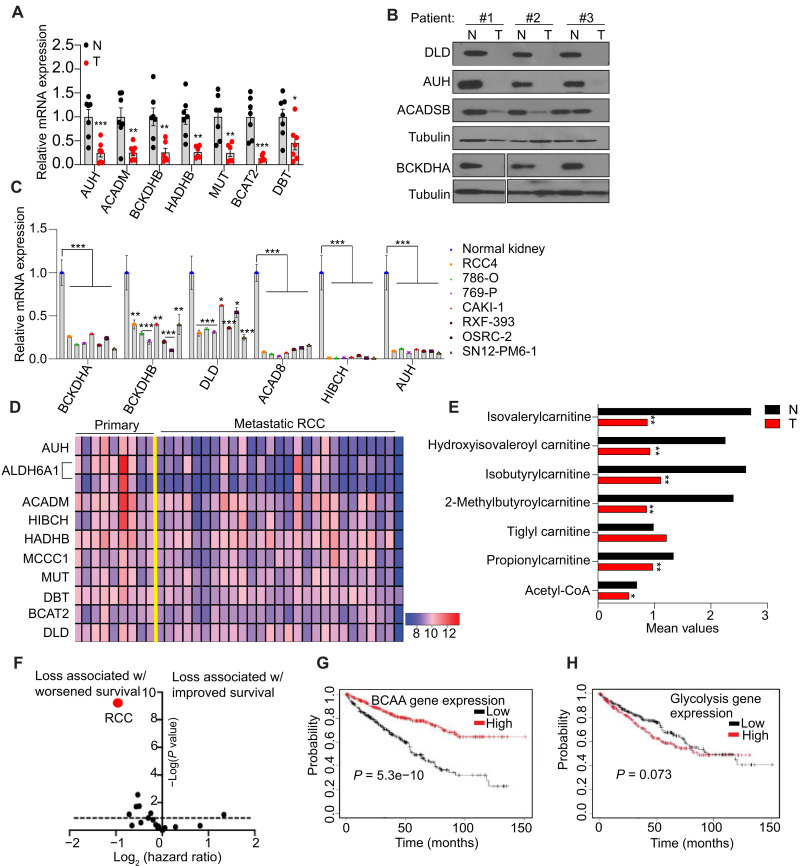
Loss of BCAA catabolic enzyme expression in renal cancer. (**A**) Relative mRNA expression of BCAA catabolic enzymes in patient-matched normal kidney (N) and tumor (T) samples (*n* = 7). Transcript levels were normalized to *RPLPO.* DBT, dihydrolipoamide branched chain transacylase E2. (**B**) Immunoblot analysis of indicated proteins in patient-matched samples. (**C**) Relative mRNA expression of BCAA catabolic enzymes in a panel of RCC cell lines relative to normal kidney (*n* = 3 to 4). (**D**) Heatmap of microarray data showing expression patterns of BCAA catabolic enzymes in primary tumor (*n* = 9) and metastatic RCC (*n* = 26). Colors represent log-transformed quantile normalized expression values. (**E**) Comparative analysis of short-chain acylcarnitine-related metabolites in 59 paired RCC tumor and normal kidney samples from metabolomic profiling. (**F**) Volcano plot showing the association between BCAA catabolic gene expressions (38 genes) and the risk of cancer death across 21 different tumor types. (**G** and **H**) Overall survival of patients with RCC as a function of BCAA (G) and glycolysis gene (H) expression. Data were extracted from TCGA RNA sequencing (RNA-seq) data on RCC using the Kaplan-Meier plotter. In (A), (C), and (E), data are presented as means ± SEM. Two-tailed Student’s *t* test was used for (A) and (E), and ordinary one-way ANOVA with Tukey’s multiple comparisons test was used for (C). *P < 0.05, **P < 0.01, and ***P < 0.001.

Prior TCGA data analysis indicated reduced mRNA expression of genes encoding BCAA catabolic enzymes in renal cancer (*5*). To validate these findings, we examined the expression of BCAA catabolic enzyme in patient-matched samples. We found reduced mRNA expression of BCAA catabolic enzymes in renal tumors compared to adjacent normal tissue (Fig. 3A). Consistent with these data, protein levels of BCAA catabolic enzymes were lower in renal tumor tissues relative to uninvolved adjacent kidney (Fig. 3B). Similarly, mRNA expression of BCAA catabolism genes is lower in RCC cell lines as compared to normal kidney (Fig. 3C). We previously reported a transcriptomic array analysis of primary ccRCC and metastatic ccRCC tumor deposits (*8*). These data reveal further decreases of BCAA catabolic enzyme mRNAs in metastatic RCC tissue compared to primary tumors (Fig. 3D). In agreement with these data, unbiased metabolomic profiling of 59 matched tumor/normal pairs revealed that short-chain carnitines derived from BCAA catabolism are significantly reduced in RCC (Fig. 3E and table S2). We next examined the prognostic significance of BCAA catabolism mRNA expression (gene list provided in table S3) in various tumor types within the TCGA dataset. Among all tumor types, loss of BCAA catabolic enzyme expression is most strongly associated with worsened survival in patients with ccRCC (Fig. 3, F and G). As a comparison, we examined the prognostic significance of glycolysis genes (gene list is provided in table S3), which are commonly increased because of alterations of the *VHL* pathway in RCC. In contrast, the expression of glycolysis enzyme mRNAs did not correlate with the patient outcome (Fig. 3H).

### The expression of BCAA catabolic enzymes is reduced in experimental models of metastatic RCC

**Fig. 4.**
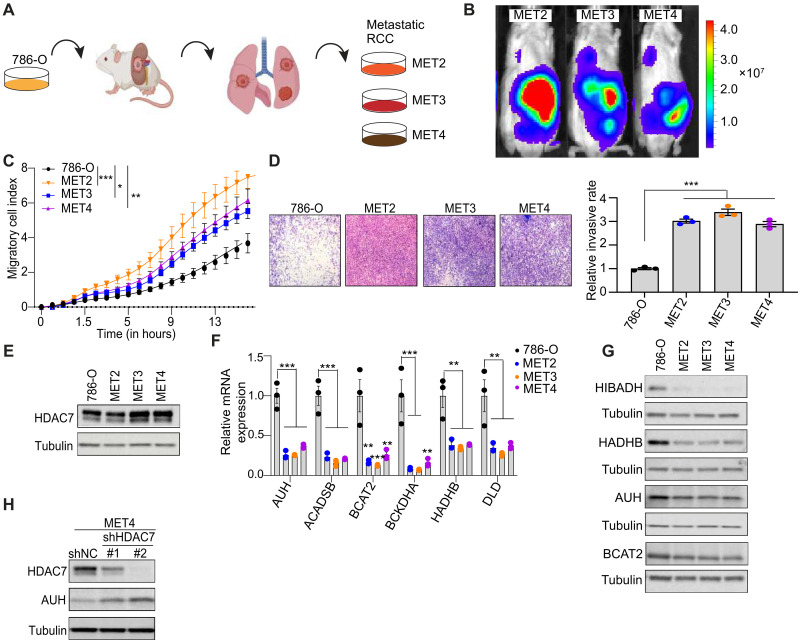
Suppression of BCAA catabolic enzymes in metastatic RCC. (**A**) Schematic diagram illustrating the generation of metastatic RCC cell lines (MET2, MET3, and MET4) from parental 786-O cells. (**B**) Luciferase-expressing 786-O cells (1.5 × 10^6^) were implanted into the renal subcapsular region of SCID mice. In vivo BL imaging of luciferase activity was performed 12 weeks postinjection. Resected lung tumors were enzymatically digested with collagenase III, and cells were then cultured in RPMI 1640 medium to establish MET2, MET3, and MET4 cell lines. (**C**) Migration kinetics of metastatic RCC cells measured using the RTCA system (*n* = 4). (**D**) Representative images and quantification of invasive cells from Boyden chamber invasion assay with Matrigel insert, comparing parental 786-O and metastatic RCC cells (*n* = 3). (**E**) Western blot analysis of HDAC7 expression in parental 786-O and metastatic RCC cells. (**F**) Relative mRNA expression of BCAA catabolic enzymes in 786-O and metastatic RCC cells. Transcript levels were normalized to *RPLPO*. (**G**) Western blot analysis of indicated proteins in 786-O and metastatic RCC cells. (**H**) Western blot analysis of indicated proteins in MET4 cells stably expressing shNC or two independent HDAC7 shRNA constructs. In (C), (D), and (F), data are presented as means ± SEM and are representative of at least two independent experiments. Ordinary one-way ANOVA with Tukey’s multiple comparisons test was used for (C), (D), and (F). *P < 0.05, **P < 0.01, and ***P < 0.001.

Given the prognostic significance of BCAA catabolism gene expression in RCC, we next examined the expression of BCAA metabolic enzymes in metastatic models of RCC. Parental 786-O cells were injected into the subcapsular region of the kidney in NOD/SCID mice. At 12 weeks postinjection, mice developed lung metastases. We dissected tumor nodules from the lungs and generated metastatic RCC cell lines (MET2, MET3, and MET4) (Fig. 4, A and B). MET cells showed no significant difference in the proliferation rate compared to parental 786-O cells (fig. S3B). However, MET cells demonstrated significantly enhanced migratory and invasive phenotypes compared to parental 786-O cells (Fig. 4, C and D). Notably, HDAC7 protein levels were higher in MET3 and MET4 cells compared to 786-O parental cells, although mRNA levels were not affected (Fig. 4E and fig. S3C). Noticeably, the mRNA expression of BCAA catabolic enzymes was significantly decreased in all MET cells (MET2, MET3, and MET4) relative to parental 786-O cells (Fig. 4F). Consistent with the mRNA findings, protein levels of BCAA catabolic enzymes were also reduced in MET cells (Fig. 4G). On the basis of these data, we performed loss-of-function studies via shRNA-mediated knockdown of *HDAC7* in MET4 cells, which exhibited higher HDAC7 expression. HDAC7 knockdown in MET4 cells resulted in increased levels of the BCAA catabolism enzyme AUH (Fig. 4H). Collectively, these data demonstrate that experimental models of RCC progression exhibit epigenetic suppression of BCAA catabolism genes.

### HDAC7 suppresses BCAA catabolism through a bimodal mechanism

**Fig. 5.**
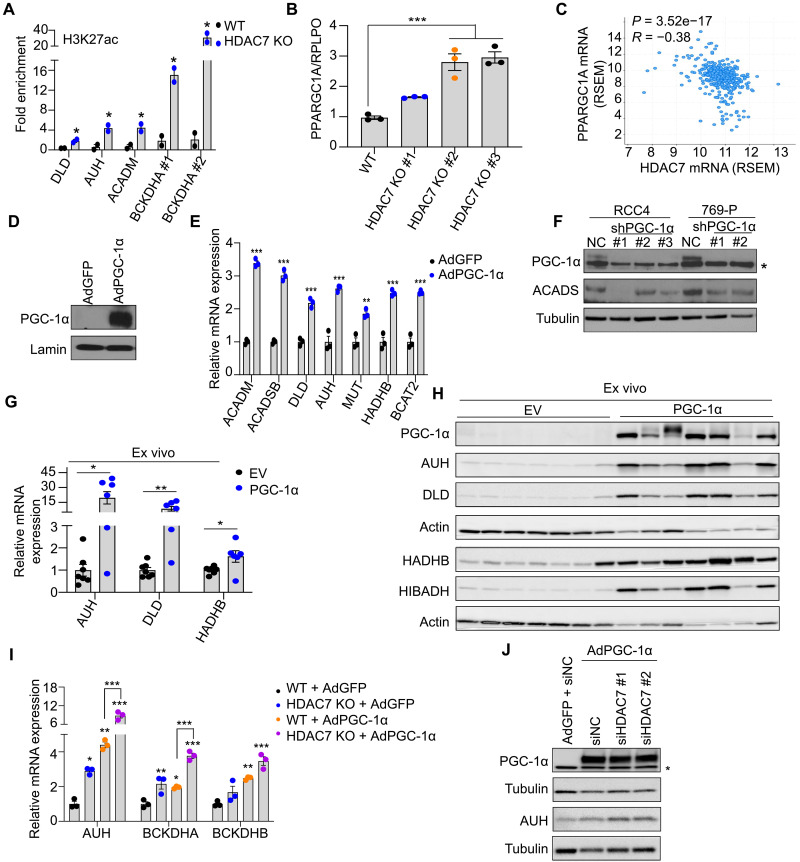
PGC-1α reexpression restores the expression of BCAA catabolic enzymes in RCC. (**A**) ChIP-qPCR was performed on 769-P cells (WT or HDAC7 KO) with rabbit immunoglobulin G and antiacetyl histone H3 (Lys^27^). The enriched DNA was quantified by qPCR (*n* = 2). (**B**) Relative mRNA expression of *PPARGC1A* in WT and three independent *HDAC7* KO 769-P cell lines (*n* = 3). RPLP0, ribosomal protein large P0. (**C**) Correlation analysis between *PPARGC1A* and *HDAC7* in TCGA KIRC dataset for renal tumors. Data were extracted using the cBioPortal web server. (**D**) Western blot analysis of PGC-1α in CAKI-1 cells expressing AdGFP or AdPGC-1α. (**E**) Relative mRNA expression of BCAA catabolic enzymes in CAKI-1 cells expressing AdGFP or AdPGC-1α (*n* = 3). (**F**) Western blot analysis of indicated proteins in RCC cells stably expressing NC or shRNA for PGC-1α. Asterisk indicates a nonspecific band. (**G** and **H**) SN12-PM6-1 cells stably expressing EV or PGC-1α were orthotopically implanted into the kidney of SCID mice. At 6 weeks postinjection, tumor-bearing kidney tissues were harvested and analyzed for mRNA (G) and protein (H) expression of the indicated genes (*n* = 7). (**I**) Relative mRNA expression of indicated genes in WT or HDAC7 KO 769-P cells. Cells were infected with AdGFP or AdPGC-1α for 72 hours. (**J**) Western blot analysis of indicated proteins in CAKI-1 cells expressing PGC-1α, transfected with either 50 nM control siRNA (siNC) or HDAC7 siRNA for 72 hours (*n* = 3). Asterisk indicates a nonspecific band. In (A), (B), (E), (G), and (I), data are presented as means ± SEM and are representative of at least two independent experiments. Two-tailed Student’s *t* test was used for (A), (E), and (G), and ordinary one-way ANOVA with Tukey’s multiple comparisons test was used for (B) and (I). *P < 0.05, **P < 0.01, and ***P < 0.001.

We next considered how HDAC7 suppresses the mRNA expression of BCAA catabolism genes. HDACs can suppress gene transcription through the local chromatin architecture at target genes (*9*). We therefore examined the impact of HDAC7 on histone 3 lysine 27 acetylation (H3K27ac) levels at BCAA catabolism gene loci using chromatin immunoprecipitation–quantitative polymerase chain reaction (ChIP-qPCR). This mark is associated with higher gene transcription. *HDAC7* ablation in RCC cells led to increased H3K27ac within promoter/enhancer regions of BCAA catabolism genes (Fig. 5A). Prior studies have shown that *PPARGC1A* expression positively correlates with the expression of BCAA catabolic genes (*10*). *PPARGC1A* encodes the transcription factor peroxisome proliferator–activated receptor gamma coactivator 1-alpha (PGC-1α). Prior studies have demonstrated that PGC-1α stimulates BCAA catabolism in muscle (*11*). We previously reported that PGC-1α/*PPARGC1A* expression is lost in RCC (*8*). Given these data, we examined HDAC7 regulation of *PPARGC1A* in RCC. HDAC7 ablation in RCC cells led to increased *PPARGC1A* mRNA (Fig. 5B). In agreement with these data, mRNA levels of *PPARGC1A* are inversely correlated with *HDAC7* mRNA in the TCGA ccRCC dataset (Fig. 5C). We then evaluated the effects of PGC-1α on BCAA catabolic enzymes. We expressed PGC-1α in CAKI-1 cells via adenoviral transduction (hereafter referred to as AdPGC-1α). Immunoblot confirmed PGC-1α protein expression (Fig. 5D). PGC-1α restoration in CAKI-1 RCC cells significantly increased mRNA levels of BCAA catabolic enzymes (Fig. 5E). In agreement with these data, PGC-1α knockdown in 769-P and RCC4, which have low but detectable PGC-1α protein levels (*8*), led to decreased ACADS protein expression (Fig. 5F). We previously evaluated the role of PGC-1α reexpression on RCC tumor progression in an orthotopic RCC model (*8*). Ex vivo analysis of orthotopic xenografts also demonstrates that PGC-1α reexpression in SN12-PM6-1 cells increases both mRNA and protein levels of BCAA catabolic enzymes (Fig. 5, G and H). Given these findings, we next examined the effect of HDAC7 KO on the expression of BCAA catabolic enzymes in RCC cells as a function of PGC-1α. HDAC7 KO cells were transiently infected with either control adenovirus green fluorescent protein (AdGFP) or AdPGC-1α. HDAC7 KO with PGC-1α restoration further increased the mRNA expression of BCAA catabolic enzymes as compared with either manipulation alone (Fig. 5I). Consistently, PGC-1α–expressing RCC cells were transfected with either a small interfering RNA negative control (siNC) or siRNA targeting HDAC7. *HDAC7* knockdown further increased the AUH protein levels (Fig. 5J). Collectively, these data support that HDAC7 suppresses the mRNA expression of BCAA catabolism genes through effects on both *PPARGC1A* levels and local effects at BCAA catabolism genes.

### BCAAs promote transcriptional levels of *SNAI1* in RCC

**Fig. 6.**
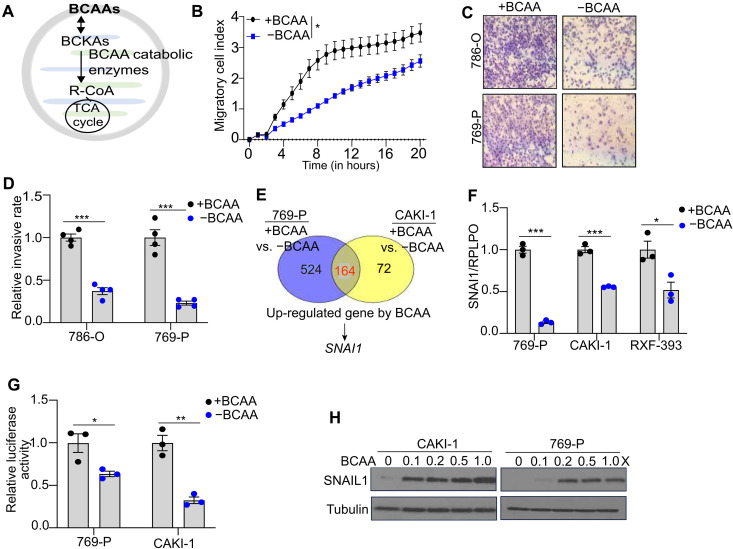
Elevated BCAA promotes transcriptional levels of SNAI1 in RCC. (**A**) Schematic diagram of BCAA metabolism. R-CoA derivatives, acyl-CoA compounds formed during the BCAA catabolism. (**B**) Migration kinetics of RXF-393 cells cultured with or without BCAA for 16 hours, measured using the xCELLigence RTCA system (*n* = 4). (**C** and **D**) Representative images (C) and quantification (D) of invasive cells from Boyden chamber invasion assay with Matrigel insert using RCC cells treated with or without BCAA (*n* = 4). (**E**) Venn diagram summarizing differently expressed genes in response to BCAA from RNA-seq data (*n* = 3). (**F**) Relative mRNA expression of SNAI1 in RCC cells cultured with or without BCAA (*n* = 3). (**G**) Luciferase activity in RCC cells transiently transfected with a firefly luciferase reporter driven by SNAI1 promoter constructs, cultured with or without BCAA (*n* = 3). (**H**) Western blot analysis of SNAIL1 in RCC cells exposed to increasing levels of BCAA for 16 hours. In (B), (D), (F), and (G), data are presented as means ± SEM and are representative of at least two independent experiments. Two-tailed Student’s *t* test was used for (B), (D), (F), and (G). *P < 0.05, **P < 0.01, and ***P < 0.001.

The BCAA catabolic pathway in the mitochondria is initiated by BCAT2, which generates BCKAs. All three BCKAs are metabolized by the BCKDH complex, followed by a series of enzymatic reactions to produce short-chain acyl-CoA species, including those entering the TCA cycle (Fig. 6A). Prior studies have shown that defective BCAA catabolism plays a critical role in increased BCAA levels in humans with obesity and type 2 diabetes (*12*). Recent findings also indicate that increased levels of BCAAs are associated with an elevated risk of certain cancers, including pancreatic and breast cancer (*13*, *14*). Our findings that the reduced BCAA catabolism gene program was strongly associated with worsened outcomes in patients with RCC led us to investigate the impact of BCAAs on aggressive phenotypes including cell invasion and migration. BCAAs significantly increased cell migration in RXF-393 cells (Fig. 6B). In addition, BCAAs markedly promoted the invasive phenotype of 786-O and 769-P cells, as determined by an invasion chamber assay with Matrigel insert (Fig. 6, C and D). The viability of RCC cells was not significantly affected by BCAA deprivation during the short time course of migration/invasion assays (~16 hours) (fig. S4).

**Fig. 7.**
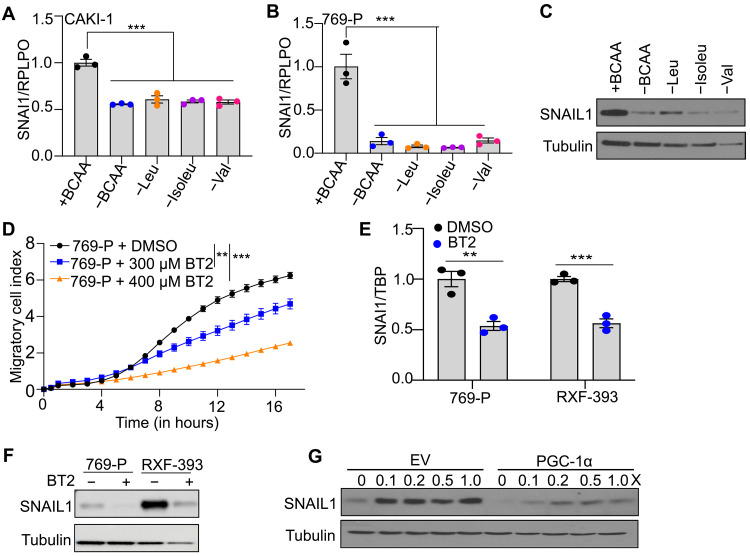
BCAA-mediated SNAIL1 expression is abolished under conditions that enhance BCAA catabolism. (**A**) Relative mRNA expression of SNAI1 in CAKI-1 cells treated without all three BCAAs or individual BCAAs (*n* = 3). (**B**) Relative mRNA expression of SNAI1 in 769-P cells treated without all three BCAAs or individual BCAAs (*n* = 3). (**C**) Western blot analysis of SNAIL1 in CAKI-1 cells treated without all three BCAAs or individual BCAAs. (**D**) Real-time migration kinetics of 769-P cells treated with BT2 were analyzed using the xCELLigence RTCA system (*n* = 4). (**E** and **F**) Relative mRNA (E) and protein (F) expression of SNAIL1 in RCC cells treated with BT2 for 24 hours. TBP, TATA box–binding protein. (**G**) Western blot analysis of SNAIL1 protein levels in CAKI-1 cells expressing EV or PGC-1α, treated with increasing concentrations of BCAA. In (A), (B), (D), and (E), data are presented as means ± SEM and are representative of at least two independent experiments. Two-tailed Student’s *t* test was used for (E), and one-way ANOVA with Tukey’s multiple comparisons test was used for (A), (B), and (D). **P < 0.01 and ***P < 0.001.

To assess BCAA’s impact on RCC biology in an unbiased manner, we performed RNA sequencing (RNA-seq) analysis on RCC cell lines (769-P and CAKI-1) cultured in the presence or absence of BCAAs. Among the most prominent changes induced by BCAA treatment in both cell lines was the increased expression of *SNAI1* (Fig. 6E). *SNAI1* encodes for SNAIL1, a master regulator of epithelial-to-mesenchymal transition (EMT) that promotes aggressive phenotypes in cancer (*15*). We further validated the increased mRNA levels of *SNAI1* by BCAA treatment via reverse transcription–qPCR (RT-qPCR) in three RCC cell lines (Fig. 6F). We then considered whether BCAAs could increase *SNAI1* mRNA levels by enhancing transcription. Using a reporter assay with the *SNAI1* promoter fused to luciferase, we found that BCAAs led to increased reporter activity (Fig. 6G). We also considered whether BCAAs could affect *SNAI1* mRNA stability. RCC cells cultured with or without BCAAs were treated with actinomycin D to block transcription. However, this led to a similar and rapid decline in *SNAI1* mRNA in both conditions (fig. S5). Consistent with mRNA data, BCAA treatment led to a dose-dependent increase in SNAIL1 protein (Fig. 6H). Although all three BCAAs have similar structures and support protein synthesis, individual BCAAs can have distinct roles (*16*). We therefore examined the effect of individual BCAAs on *SNAI1* mRNA expression. SNAIL1 mRNA and protein levels were significantly decreased in media lacking all three BCAAs. Furthermore, depleting just one BCAA led to similar effects on SNAIL1 mRNA and protein, indicating that the presence of all three BCAAs is required for optimal SNAIL1 expression in RCC (Fig. 7, A to C). Given these data, we next considered the impact of BCAA catabolism on aggressive phenotype and SNAIL1 expression. The BCKDH complex is the rate-limiting step for BCAA catabolism and is negatively regulated by the BCKDH kinase (BCKDK) (*17*). The BCKDK small molecule inhibitor 3,6-dichlorobenzo(b)thiopene-2-2-carboxylic acid (BT2) promotes the BCAA catabolic pathway via increased activation of the BCKDH complex (*18*). RCC cells treated with BT2 led to reduced migratory phenotype in 769-P cells (Fig. 7D). RCC cells treated with BT2 also showed decreased mRNA and protein expression of SNAIL1 (Fig. 7, E and F). Given that PGC-1α restoration in RCC cells increases BCAA catabolic enzymes, we evaluated its role in BCAA-mediated SNAIL1 expression. PGC-1α restoration dampened the induction of SNAIL1 by BCAA treatment (Fig. 7G). Together, these data support that BCAAs promote SNAIL1 expression in RCC, but these effects are diminished by conditions that enhance BCAA catabolism.

### Blocking BCAT2 activity rescues SNAIL1 expression in HDAC7 KO cells

**Fig. 8.**
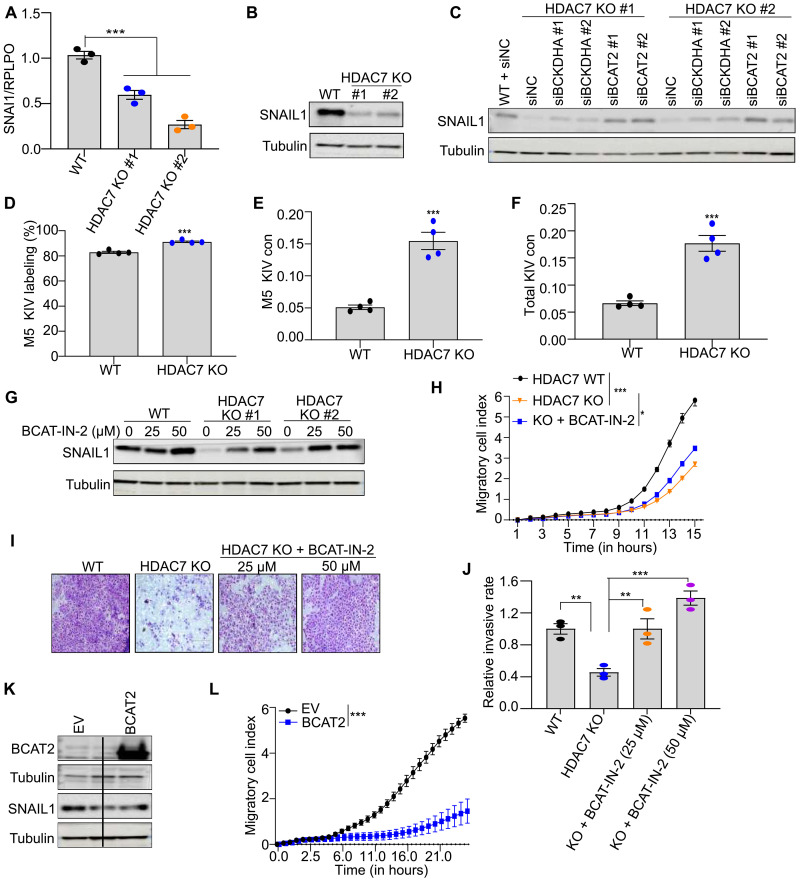
BCAT2 inhibition restores SNAIL1 expression in HDAC7 KO cells. (**A** and **B**) Relative mRNA (A) and protein (B) levels of SNAIL1 in WT and two independent HDAC7 KO 769-P cells. (**C**) Immunoblot analysis of SNAIL1 in WT and HDAC7 KO 769-P cells. HDAC7 KO cells were transfected with siNC or indicated siRNAs for 72 hours. (**D**) Fractional percent labeling of M5 KIV from [U-^13^C_5_] valine in WT and HDAC7 KO 769-P cells (*n* = 4). (**E** and **F**) Relative M5 KIV (E) and total KIV (F) concentration in WT and HDAC7 KO 769-P cells. (**G**) Western blot analysis of SNAIL1 in WT and HDAC7 KO 769-P cells treated with indicated concentrations of BCAT-IN-2 for 48 hours. (**H**) Migration kinetics of WT and HDAC7 KO 769-P cells treated with or without BCAT-IN-2, measured by the RTCA system (*n* = 4). (**I** and **J**) Boyden chamber invasion assay with Matrigel insert using WT and HDAC7 KO 769-P cells treated with or without BCAT-IN-2. Representative images (I) and quantification of invasive cells (J) are shown. (**K**) Western blot analysis of indicated proteins in 786-O cells stably expressing EV or BCAT2. Line indicates noncontiguous lanes from the same blot. (**L**) Migration kinetics of 786-O cells expressing EV or BCAT2, measured using the RTCA system (*n* = 4). In (A), (D), (E), (F), (H), (J), and (L), data are presented as means ± SEM and are representative of at least two independent experiments. Two-tailed Student’s *t* test was used for (D), (E), (F), and (L), and ordinary one-way ANOVA with Tukey’s multiple comparisons test was used for (A), (H), and (J). *P < 0.05, **P < 0.01, and ***P < 0.001.

Given that HDAC7 represses the expression of BCAA catabolic enzymes, we assessed HDAC7’s role in *SNAI1* mRNA expression. HDAC7 KO led to reduced mRNA and protein levels of SNAIL1 (Fig. 8, A and B). In concert with these data, *SNAI1* mRNA is positively correlated with *HDAC7* mRNA in the ccRCC TCGA dataset (fig. S6). We next determined whether HDAC7 suppression of BCAA catabolism is critical for its promotion of SNAIL1 in RCC. We performed loss-of-function studies via siRNA-mediated knockdown of either BCKDHA or BCAT2 in HDAC7 KO cells generated in 769-P RCC cells. Real-time RT-qPCR data demonstrating knockdown efficiency are provided in fig. S7 (A and B). Consistent with prior data, HDAC7 KO clones (#1 and #2) treated with negative control siRNA (siNC) had low SNAIL1 as compared with HDAC7 wild-type (WT) cells treated with siNC (Fig. 8C, compare lanes 2 and 7 with lane 1). BCKDHA knockdown in HDAC7 KO cells led to a partial rescue of SNAIL1. Knockdown of BCAT2, which catalyzes the first step of BCAA catabolism, led to a more pronounced rescue of SNAIL1 expression in HDAC7 KO cells (Fig. 8C). This pronounced rescue of SNAIL1 led us to examine the flux through BCAT2 as a function of HDAC7 in RCC. WT and HDAC7 KO cells were cultured with uniformly labeled [U-^13^C_5_] valine, and labeling of the ketoacid of valine, α-ketoisovalerate (KIV), was quantified via liquid chromatography–mass spectrometry. HDAC7 KO resulted in increased label incorporation into KIV, as determined by the fraction of M + 5 KIV labeling (Fig. 8D). In addition, HDAC7 KO led to increased levels of both M + 5 KIV and total KIV (Fig. 8, E and F). These data are consistent with increased flux through BCAT2 with HDAC7 ablation.

We next determined the effects of the pharmacologic BCAT2 inhibitor using BCAT-IN-2 (*19*). Treatment with BCAT-IN-2 rescued SNAIL1 protein in two distinct HDAC7 KO clones (Fig. 8G). Consistently, BCAT2 inhibition rescued migratory and invasive behaviors in HDAC7 KO cells (Fig. 8, H to J). Furthermore, ectopic BCAT2 expression in RCC cells reduced SNAIL1 levels and attenuated migration compared to control RCC cells (Fig. 8, K and L). Collectively, these data demonstrate that HDAC7 KO suppresses SNAIL1 expression in a BCAT2-dependent manner.

### Increased BCAA levels regulate *SNAI1* expression via NOTCH signaling

**Fig. 9.**
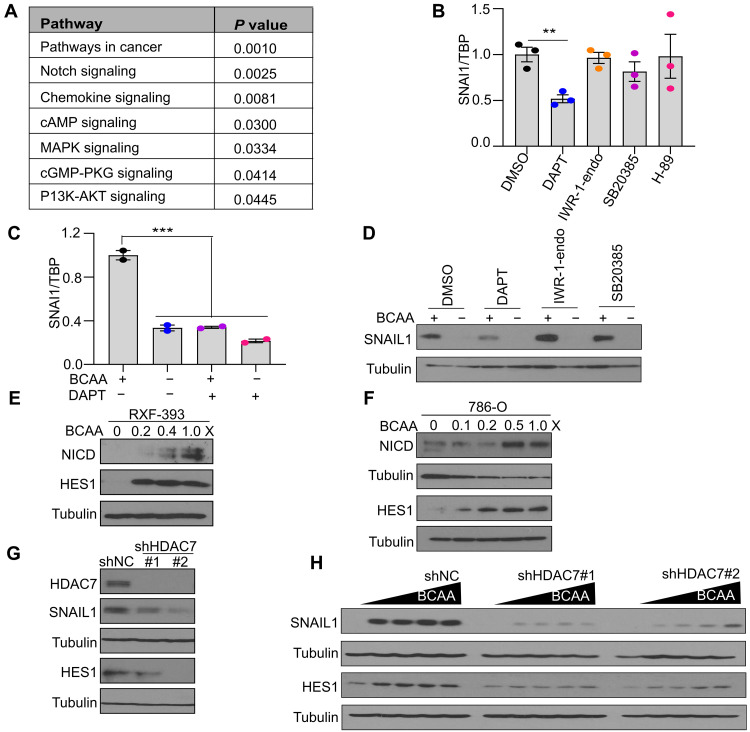
Elevated BCAA levels regulate SNAIL1 expression by NOTCH signaling. (**A**) KEGG pathway analysis showing pathways positively correlated with *SNAI1* expression in metastatic RCC samples (*n* = 26). MAPK, mitogen-activated protein kinase; cAMP, cyclic adenosine 3′,5′-monophosphate; cGMP, guanosine 3′,5′-monophosphate; PKG, cGMP-dependent protein kinase. (**B**) Relative mRNA expression of *SNAI1* in 769-P cells treated with indicated inhibitors for 48 hours. (**C**) Relative mRNA expression of *SNAI1* in 769-P cells incubated with or without 25 μM DAPT. DAPT-treated cells were exposed to media with or without BCAA for 16 hours (*n* = 3). (**D**) Immunoblot analysis of SNAIL1 expression in 769-P cells treated with specific inhibitors, followed by exposure to media with or without BCAA for 16 hours. (**E** and **F**) Western blot analysis of indicated proteins in RXF-393 cells (E) and 786-O cells (F) treated with increasing concentrations of BCAA for 16 hours. (**G**) Western blot analysis of indicated proteins in 786-O cells expressing shNC or two independent shRNA targeting *HDAC7*. (**H**) Western blot analysis of indicated proteins in 786-O cells expressing shNC or two independent shRNA targeting HDAC7, treated with increasing concentrations of BCAA for 16 hours. In (B) and (C), data are presented as means ± SEM and are representative of at least two independent experiments. Ordinary one-way ANOVA with Tukey’s multiple comparisons test was used for (B) and (C). **P < 0.01 and ***P < 0.001.

Given these data, we next investigated signaling mechanisms by which BCAAs promote SNAIL1. As an unbiased approach, we analyzed gene expression data of metastatic RCC tissues previously reported (*20*). The NOTCH pathway is among the pathways most strongly associated with *SNAI1* mRNA expression (Fig. 9A). We therefore treated RCC cells with inhibitors of various signaling pathways: NOTCH (DAPT), Wnt (IWR-1-endo), mitogen-activated protein kinase (SB20385), and cAMP-dependent protein kinase (H-89). Inhibition of NOTCH signaling significantly decreased mRNA expression of *SNAI1* (Fig. 9B). Furthermore, inhibition of NOTCH signaling with DAPT abrogated BCAA-mediated *SNAI1* mRNA and protein expression (Fig. 9, C and D). The canonical NOTCH pathway is initiated by two ligand-mediated proteolysis events that release the NOTCH intracellular domain (NICD). The NICD then translocates to the nucleus, where it transcriptionally up-regulates NOTCH target genes (e.g., HES1). BCAAs promoted a dose-dependent increase in NICD levels, a canonical readout of NOTCH activation (Fig. 9, E and F). We also observed that BCAA treatment led to increased levels of the NOTCH target gene HES1 (Fig. 9, E and F). Collectively, these data support that HDAC7-mediated suppression of BCAA catabolism results in BCAA-mediated activation of NOTCH signaling, which, in turn, transcriptionally up-regulates SNAIL1. In agreement, HDAC7 knockdown reduced protein levels of both SNAIL1 and NOTCH target gene HES1 (Fig. 9G). Last, we assessed the impact of BCAAs on SNAIL1 expression as a function of HDAC7. BCAA induction of SNAIL1 was diminished in *HDAC7* knockdown cells. Furthermore, BCAA-mediated induction of HES1 (NOTCH target gene) was markedly diminished in HDAC7 knockdown cells (Fig. 9H).

## DISCUSSION

Metabolic reprogramming is recognized as a hallmark of cancer cells. Among various tumor types, ccRCC demonstrates a metabolic switch toward enhanced glycolysis (*21*). Mutational inactivation of *VHL* leads to constitutive stabilization of hypoxia-inducible factor, which promotes the transcription of glycolysis genes. While current understanding of RCC tumor biology primarily focuses on the reprogramming of glucose metabolism, our findings indicate that there are additional metabolic alterations with implications for tumor progression including the loss of BCAA catabolism. Despite the focus on glycolysis in ccRCC, the expression of glycolysis genes shows no association with patient outcome. In contrast, loss of BCAA catabolism genes is strongly associated with worsened outcome in patients with ccRCC as compared to all other tumor types in the TCGA cohort. These data prompted us to investigate the molecular basis underlying altered BCAA catabolism in ccRCC and its role in tumor progression.

HDAC7, a member of class IIa HDACs, is known to mediate transcriptional repression (*22*, *23*). Elevated expression of HDAC7 has been observed in various malignant tumors, glioma, colorectal, and gastric cancers (*24*–*27*). However, insight into the functional significance of HDAC7 expression on tumor progression has been limited to date. Here, we demonstrate the role of HDAC7 in tumor progression using an orthotopic tumor model.

We previously implicated HDAC7 in suppressing TCA cycle enzymes in RCC (*6*). In the present study, we elucidate a role for HDAC7 in the metabolic remodeling of BCAA metabolism. Alterations in BCAAs and their metabolism have been implicated in cancer. While previous studies have reported that the transcript levels of BCAA catabolic enzymes are down-regulated in ccRCC (*5*, *28*), the molecular mechanisms underlying this defective BCAA catabolism and its consequent role in RCC tumor progression were not studied.

Our data support a bimodal mechanism by which HDAC7 suppress the BCAA catabolism program. First, our data demonstrate that HDAC7 suppresses *PPARGC1A*, which encodes PGC-1α. We previously demonstrated that *PPARGC1A* expression is lost in metastatic RCC (*8*). Our studies support a mechanism for the suppression of BCAA catabolic enzymes through *PPARGC1A* loss. In addition, our data support that HDAC7 also has direct effects on the chromatin architecture of BCAA catabolism genes.

While altered BCAA catabolism has been implicated in tumor cell proliferation, its role in promoting aggressive phenotypes that drive tumor progression/metastasis is less well characterized. Our findings reveal a connection between remodeled amino acid metabolism and SNAIL1 expression. Prior studies have demonstrated that increased SNAIL1 protein expression is associated with worsened prognosis in RCC (*29*). SNAIL1 plays a crucial role in promoting metastasis in response to several oncogenic signaling pathways such as transforming growth factor–β and Wnt signaling (*30*, *31*). Our data indicate that the remodeling of BCAA catabolism promotes SNAIL1 expression through NOTCH signaling. NOTCH signaling has been shown to promote EMT and metastasis in various cancers, including breast, colorectal, and pancreatic cancers (*32*–*34*). Recent studies have also implicated the NOTCH pathway in ccRCC (*35*).

Our studies point to several potential therapeutic approaches to mitigate tumor progression by targeting the outlined axis. This is highly relevant, given that direct approaches to target SNAIL1 have proven challenging. HDAC7 could be targeted by broad HDAC inhibitors, some of which are now in clinical trials. More recent studies demonstrate the feasibility of targeting HDAC7 specifically via a proteolysis targeting chimera approach (*36*). Alternatively, pharmacologic inhibition of BCKDK could be adopted to promote BCAA catabolism. A recent clinical trial demonstrated that the BCKDK inhibitor sodium phenylbutyrate can lower plasma BCAA levels in patients with type 2 diabetes (*37*). An agent that promotes BCAA catabolism could have multiple benefits. Enhancing BCAA catabolism in peripheral tissues could reduce BCAA availability for tumor cells. In addition, our data support that activating BCAA catabolism within tumor cells could mitigate aggressive phenotypes. An alternative approach would be strategies to promote BCAT2 activity or its expression could also be deployed to promote BCAA catabolism in RCC cells.

There are some limitations to our study. Although the data presented support a role for HDAC7’s effects on BCAA catabolism, they do not exclude alternate effects of HDAC7 on the expression of genes that affect tumor progression. In addition, the precise mechanism by which the remodeling of BCAA catabolism leads to NOTCH-mediated SNAIL1 expression is unclear. With regard to signaling, BCAAs have primarily been implicated in mechanistic target of rapamycin activation. Hence, the signaling cascades activated (or blocked) by the remodeling of BCAA metabolism warrant further investigation based on our findings.

**Fig. 10.**
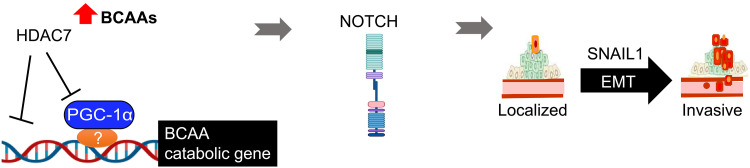
A proposed working model for HDAC7-mediated RCC metastasis. HDAC7-mediated suppression of BCAA catabolism results in BCAA-dependent activation of NOTCH signaling, which subsequently up-regulates SNAI1 transcription.

In summary, we report that HDAC7 promotes the remodeling of BCAA catabolism (Fig. 10). In turn, this remodeling promotes transcription of SNAIL1, a key factor that promotes aggressive behaviors in RCC. These findings provide insight into the HDAC7’s role as a transcriptional repressor that promotes RCC progression. Hence, our findings provide compelling evidence that HDAC7 or its metabolic sequelae could be targeted to block tumor progression in one of the most common malignancies.

## MATERIALS AND METHODS

### Cell culture

RCC cell lines (786-O, 769-P, CAKI-2, CAKI-1, A498, and OSRC2) and HK-2 renal epithelial cells were obtained from the American Type Culture Collection. RCC4 cells were provided by P. Ratcliffe (Oxford University, Oxford, England), and SN12-PM6-1 cells were provided by R. S. Kerbel (Sunnybrook Research Institute, Toronto, Canada). RXF-393 cells were obtained from the National Cancer Institute (NCI). 786-O, 769-P, RXF-393, and OSRC2 were cultured in RPMI 1640 medium (Corning Life Science) supplemented with 10% fetal bovine serum (FBS; R&D Systems) with penicillin-streptomycin (100 U/ml). CAKI-1 and CAKI-2 cells were maintained in MEM (minimum essential medium) medium supplemented with 10% FBS and antibiotics. HK-2 and RCC4 were cultured in Dulbecco’s modified Eagle’s medium. All cell lines were periodically screened for mycoplasma contamination using a PCR-based mycoplasma detection kit (MilliporeSigma).

### Chemicals

To generate BCAA-depleted culture medium, amino acid–free RPMI 1640 powder (United States Biological Life Sciences) was reconstituted and supplemented with 10% dialyzed FBS (Cytiva). RCC cells were treated with BCAA (0.38 mM isoleucine, 0.38 mM leucine, and 0.17 mM valine). BT2 was purchased from Matrix Scientific. The γ-secretase inhibitor DAPT was obtained from MilliporeSigma. IWR-1-endo, SB20385, and H-89 were purchased from Selleck Chemicals. BCAT-IN-2 was purchased from MedChemExpress.

### Plasmid and virus infections

Lentiviral shRNA constructs for HDAC7 and PPARGC1A were purchased from MilliporeSigma. Recombinant lentivirus particles were generated by cotransfecting lentivirus plasmid vectors with packaging and envelope plasmids into human embryonic kidney 293T cells using calcium phosphate–mediated transient transfection. Detailed experimental procedures are previously described (*8*). The virus-containing supernatant was harvested 72 hours posttransfection and concentrated using the Lenti-X concentrator (Clontech). RCC cells were then infected with the viral supernatant in the presence of polybrene (8 μg/ml), followed by antibiotic selection.

### Generation of HDAC7 KO cell lines

HDAC7 KO cell lines were generated using CRISPR-Cas9 technology. CRISPR guide RNAs (gRNAs) targeting human HDAC7 were designed using the CHOPCHOP design tool (https://chopchop.cbu.uib.no/) (*38*). The selected gRNA sequences were as follows: exon 6, 5′-CGGGCGTGCTGCTACTACTT-3′; exon 2, 5′-CTCGGGCATCGGCGTGTCCA-3′; and exon 3, 5′-TAGGGAATGCCGGGGCTGTT-3′. These single gRNAs were cloned into the lentiCRISPRv2 vector using the Bsm BI cloning site, as previously described (*39*).

### siRNA transfection

RCC cells were transiently transfected with 50 nM of either an NC or the indicated specific siRNA using Lipofectamine RNAiMAX reagent (Invitrogen) for 72 hours. Transfections were performed according to the manufacturer’s protocol.

### Gene expression profiling in patients with renal cancer

Gene expression profiling data on RCC tissues have been previously reported and are accessible at National Center for Biotechnology Information Gene Expression Omnibus

(GEO) GSE105288 (*20*). BCAA catabolic gene expression was analyzed using patient-matched RCC tumors and adjacent normal tissue in a separate cohort (*n* = 7). Tissues were acquired from the Cooperative Human Tissue Network. Detailed sample collection procedures and patient information were described previously (*20*). All tissues are deidentified.

### Kaplan-Meier plotter database analysis and correlation analysis

The Kaplan-Meier plotter tool was used to analyze the prognostic value of BCAA and glycolysis gene expression (www.kmplot.com/analysis). Inverse correlations between HDAC7 and BCAA catabolic enzymes were analyzed using the TCGA KIRC dataset through cBioPortal web server (www.cbioportal.org).

### Doubling time and cell viability assays

For a doubling time assay, 769-P WT and HDAC7 KO cells were seeded in 60-mm dishes and cultured for 3 days. Doubling times were calculated using the formula: *doubling time* = (*duration* × ln)/[ln(*X*e/*X*b)] (*2*), where Xe represents the cell count at the end of the incubation period, and Xb represents the initial cell count. To assess cell viability, RCC cells were seeded in 96-well plates. After 48 hours, cells were cultured in media with or without BCAA for 16 hours. Cell viability was then measured using the MTT assay (Abcam, ab211091) according to the manufacturer’s protocol.

### Migration and invasion assays

Cell migration rates were measured in real time using the xCELLigence system (Agilent) with Cell Invasion and Migration plate. For Boyden chamber invasion assays, Corning BioCoat Matrigel Invasion Chambers were used. RCC cells were seeded onto cell culture inserts, coated with a layer of Matrigel, and allowed to invade for 16 hours toward 10% FBS in the bottom wells. Invaded cells were stained using the Diff-Quik Stain Kit (Siemens) and counted under a light microscope.

### RNA isolation and RT-qPCR

Total RNA from RCC tissues was isolated using the RNeasy Mini Kit (QIAGEN). For cultured cells, total RNA was extracted using TRIzol reagent (Ambion) according to the manufacturer’s instructions. cDNA synthesis was performed using the High-Capacity cDNA Reverse Transcription Kit (Thermo Fisher Scientific). TaqMan qPCR assay was conducted using a QuantStudio 6k Flex Real-Time PCR system (Thermo Fisher Scientific). The TaqMan probes used for RT-qPCR are listed in table S4. mRNA expression of targeted genes was normalized to the expression of ribosomal protein large P0. Relative changes in gene expression from RT-qPCR were analyzed using the comparative cycle threshold (CT) method (2^−ΔΔ**CT**^).

### Luciferase assay

Snail-pGL2 Snail promoter luciferase reporter plasmid (#31694) and Renilla plasmid (pRL-SV40P, #27163) were obtained from Addgene. Cells were transfected for 48 hours with Lipofectamine and then cultured in media containing 10% dialyzed FBS with or without BCAAs for 16 hours. After cell counting, cells were seeded in a 96-well plate, and then Firefly luciferase and Renilla luciferase (internal control) activities were measured using the Promega Dual-Glo Luciferase Assay (Thermo Fisher Scientific).

### Chromatin immunoprecipitation–quantitative polymerase chain reaction

The ChIP assay was performed using the EZ-Magna ChIP Chromatin IP A/G kit (MilliporeSigma) according to the manufacturer’s instructions. RCC cells were cross-linked with 1% formaldehyde for 10 min and quenched with glycine for 5 min. Chromatin was sheared by sonication using the Bioruptor Pico (Diagenode). ChIP experiments were performed using anti-H3K27ac antibody and antirabbit immunoglobulin G antibody. After pull-down, input DNA and immunoprecipitated DNA were purified with the DNA Clean and Concentrator kit (Zymo Research). Target DNA enrichment was calculated using the fold enrichment method. Primer sequences for ChIP-qPCR are described in table S5.

### Untargeted metabolomic analysis

Tissues from patient-matched RCC tumors and adjacent normal tissues (*n* = 59) were acquired from the Cooperative Human Tissue Network. Untargeted metabolomic analysis was conducted by Metabolon Inc. (Durham, NC, USA). Metabolites were extracted with methanol, and the detailed protocol was described previously (*40*).

### RNA sequencing

Total RNA was extracted from RCC cells using the RNeasy Mini Kit (QIAGEN). Subsequent RNA sample processing and sequencing analysis services were provided by HudsonAlpha Institute. The gene expression profiling data have been deposited at GEO (#GSE291765).

### Immunoblotting analysis

RCC cells were lysed with SDS buffer containing 1× protease and phosphatase inhibitor cocktail (Thermo Fisher Scientific). Protein concentrations were determined using the RC DC Protein Assay (Bio-Rad). Denatured protein samples in Laemmli buffer were separated electrophoretically on SDS–polyacrylamide gels and transferred to polyvinylidene difluoride membranes. Blots were incubated with primary antibodies for overnight at 4°C on a rocker. After washing, blots were incubated with horseradish peroxidase–conjugated secondary antibodies (Amersham Biosciences). Immunoreactivity was visualized using enhanced chemiluminescence (SuperSignal West Dura, Pierce). Antibodies used in this study are described in table S6.

### ^13^C_5_ valine incorporation analysis

WT and HDAC7 KO 769-P cells were cultured to ~80% confluence. Cells were then incubated in secretion buffer containing 170 μM [^13^C_5_] valine (MilliporeSigma) for 30 min (*41*). Following incubation, cells were rinsed with phosphate-buffered saline. Metabolite extraction was performed using ice-cold methanol containing 0.05 mM l-norvaline as an internal standard. A second methanol extraction was carried out, and both methanol fractions were combined. The combined extracts were centrifuged at 16,000*g* for 15 min. After centrifugation, the supernatant was dried under vacuum. The resulting pellets were reconstituted in a solvent mixture (water/methanol/chloroform, 1:1:1, v/v), and the reconstituted samples were analyzed by gas chromatography–mass spectrometry, as previously described (*42*).

### Animals

Male SCID/NOD mice were purchased from Charles River Laboratories (Wilmington, MA, USA) at 5 to 6 weeks of age. All mice were housed under a 12:12-hour light-dark cycle in ventilated cages. All animal procedures were approved by the Institutional Animal Care and Use Committee at the University of Alabama at Birmingham (#22647).

### Generation of orthotopic renal cancer mouse models

Mice were anesthetized using a ketamine-xylazine cocktail. A vertical incision through the left flank was made to expose the lateral aspect of the kidney. Luciferase-expressing RCC cells (1.5 × 10^6^ cells) were mixed with Matrigel in a 1:1 ratio and injected under the renal capsule into the lower pole using a Hamilton syringe. Tumor progression was monitored weekly by live-animal bioluminescence optical imaging using an IVIS Lumina III imaging system (PerkinElmer, Hopkinton, MA, USA). To produce bioluminescence, mice were intraperitoneally injected with d-luciferin stock solution (150 mg/kg).

### Isolation of metastatic RCC cells from orthotopic animal models

Parental 786-O cells were stably transfected with a luciferase reporter plasmid and selected with G418/geneticin (400 μg/ml). Luciferase-expressing 786-O cells (1.5 × 10^6^ cells) were injected under the renal capsule of male SCID/NOD mice. At 12 weeks postinjection, tumor nodules from the lungs were harvested and enzymatically digested with 0.125% collagenase III and 0.1% hyaluronidase. Samples were gently rocked at 37°C for 4 hours, briefly centrifuged, and resuspended in 0.25% trypsin. Cells were then centrifuged and cultured in RPMI 1640 medium with G418/geneticin.

### Statistical analysis

For in vitro studies, data are presented as means ± SEM from at least two to three independent experiments. The exact number of samples is described in each figure legend. The two-tailed Student’s *t* test was used to assess significant differences between the means of two groups. One-way analysis of variance (ANOVA) was used to determine statistically significant differences between the means of three or more independent groups.
