# Supplementary material for: HDAC7 promotes renal cancer progression by reprogramming branched-chain amino acid metabolism
Source: Sci Adv. 2025 Jun 4;11(23):eadt3552. doi: 10.1126/sciadv.adt3552 (PMC12136004; doi:10.1126/sciadv.adt3552)
Supplement: Supplementary file 1 — Figs. S1 to S7 Tables S1 to S6 [file sciadv.adt3552_sm.pdf]

Science Advances
AAAS

Supplementary Materials for

HDAC7 promotes renal cancer progression by reprogramming branched-chain amino acid metabolism

Hyeyoung Nam *et al.*

Corresponding author: Sunil Sudarshan, sudarshan@uab.edu

Sci. Adv. **11**, eadt3552 (2025)
DOI: 10.1126/sciadv.adt3552

This PDF file includes:

Figs. S1 to S7
Tables S1 to S6

SUPPLEMENTAL DATA

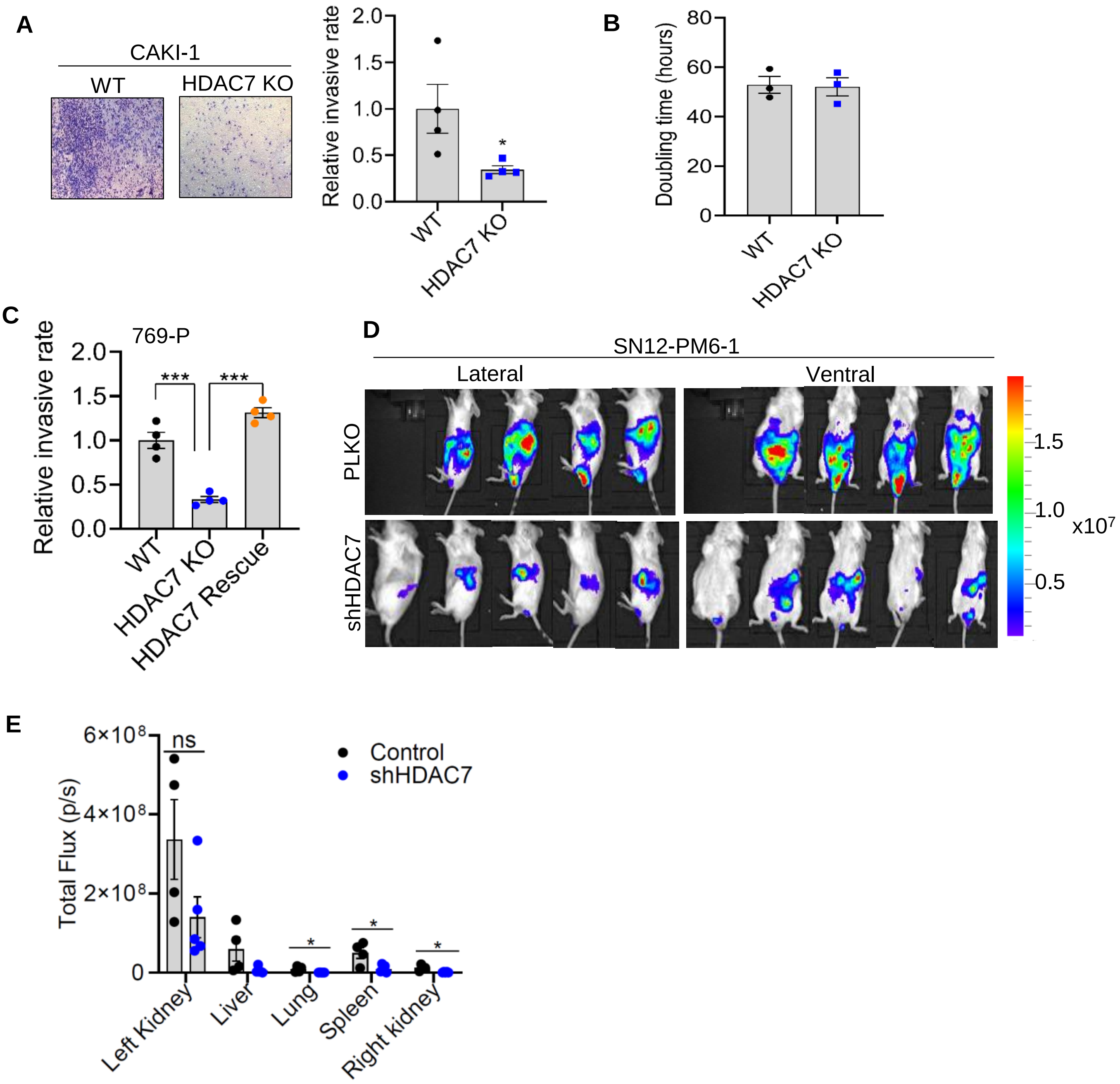

Figure S1. HDAC7 promotes RCC metastasis. (**A**) Representative images and quantification of invasive cells from Boyden chamber invasion assay with Matrigel insert using WT or HDAC7 KO CAKI-1 cells. (**B**) Average doubling time of HDAC7 WT and KO cells. (**C**) Quantification of invasive cells from Boyden chamber invasion assay with Matrigel insert using 769-P WT, HDAC7 KO, and HDAC7 rescue cells (n = 4). (**D**) Luciferase-expressing SN12-PM6-1 cells were orthotopically implanted into the kidney capsule of SCID mice. Bioluminescence was used to monitor tumor metastasis at 8 weeks post-injection (n = 4 ~ 5). (**E**) Quantification of *ex vivo* BL signals in tissues from mice orthotopically implanted with SN12-PM6-1 cells. In (A), (B), (C), and (E) data are presented as means ± SEM. Two-tailed Student's *t* test was used for (A), (B), and (E), and ordinary one-way ANOVA with Tukey's multiple comparison test was used for (C). * $P < 0.05$; *** $P < 0.001$.

SUPPLEMENTAL DATA

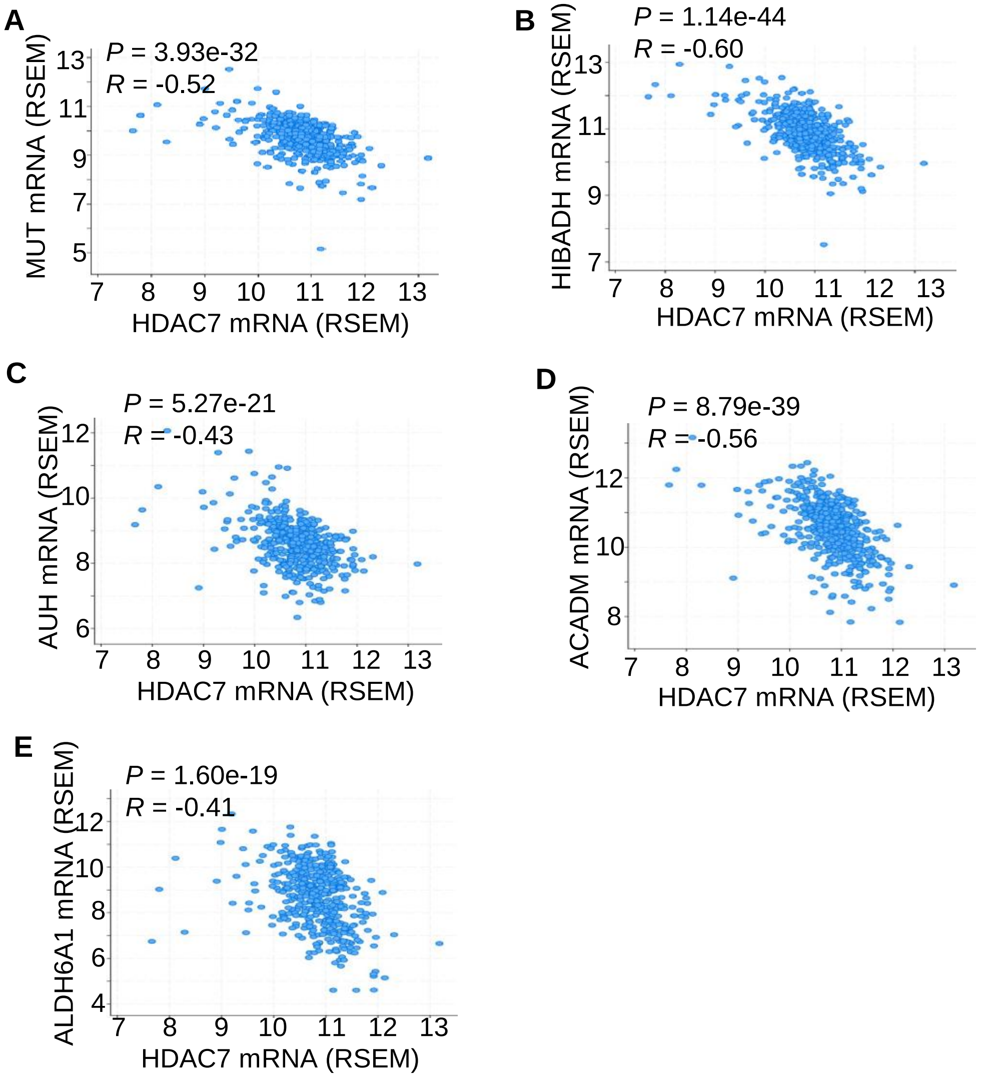

Figure S2. The expression of BCAA catabolic genes is negatively correlated with the expression of *HDAC7* in ccRCC. (A-E) Results of negative correlation analysis between the expression of BCAA catabolic genes (*MUT, HIBADH, AUH, ACADM*, and *ALDH6A1*) and *HDAC7* in RCC samples from TCGA KIRC data set.

SUPPLEMENTAL DATA

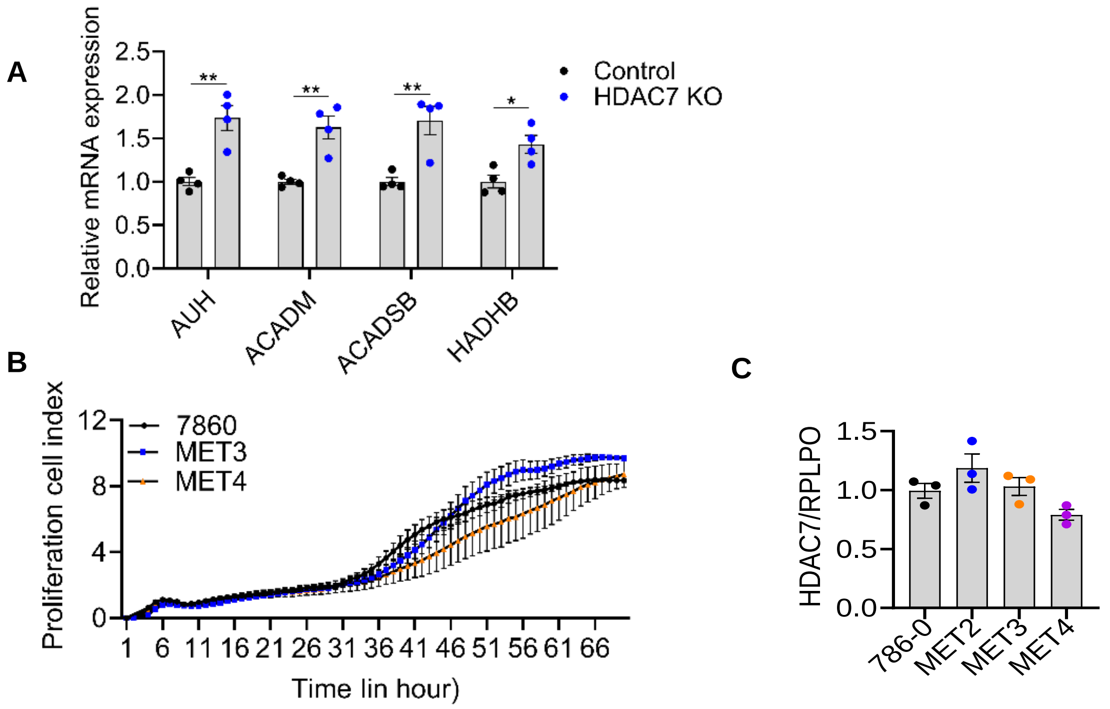

Figure S3. Characterization of RCC models with altered expression of BCAA catabolic enzymes. (A) Relative mRNA expression of BCAA catabolic genes in CAKI WT and *HDAC7* KO cells (n = 4). (**B**) Proliferation rate in MET and 786-0 cells measured by the RTCA system (n = 4). (**C**) Relative mRNA expression of *HDAC7* in MET and 786-0 cells (n =3). In (A), data are presented as means ± SEM. Two-tailed Student's *t* test was used for (A). * $P < 0.05$; ** $P < 0.01$.

SUPPLEMENTAL DATA

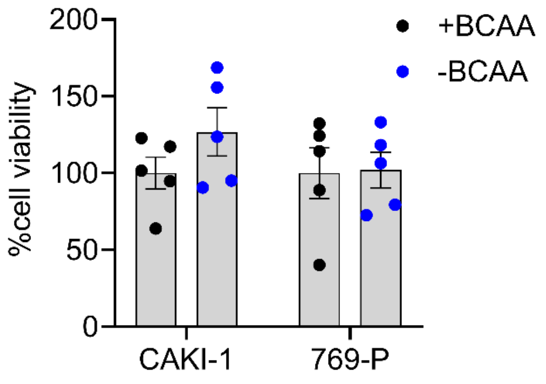

Figure S4. Effect of BCAA deprivation on cell viability. RCC cells were treated without or with BCAA for 16 hours. MTT assay was performed to measure cell viability (n = 5).

SUPPLEMENTAL DATA

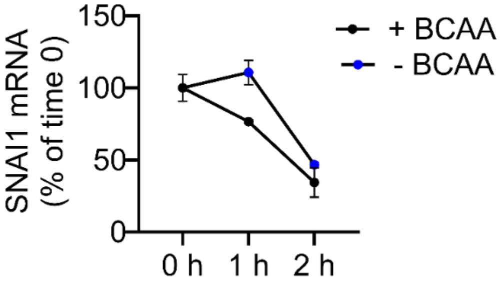

Figure S5. BCAAs do not affect mRNA stability of *SNAI1* in RCC cells. RXF-393 cells were exposed to in the presence or absence of BCAA for 16 hours and then treated with actinomycin D at the indicated time points (n = 2).

SUPPLEMENTAL DATA

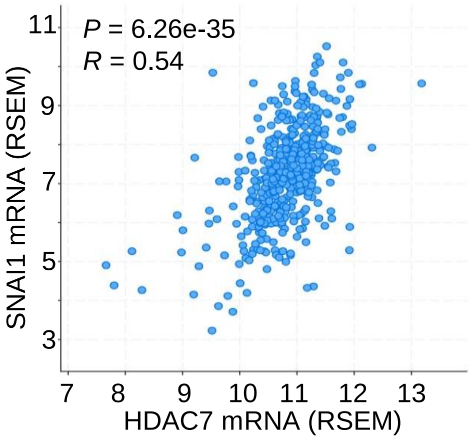

Figure S6. The expression of *SNAI1* is positively correlated with the expression of *HDAC7* in ccRCC. Results of positive correlation analysis between the expression of *SNAI1* and *HDAC7* in RCC samples from TCGA KIRC data set.

SUPPLEMENTAL DATA

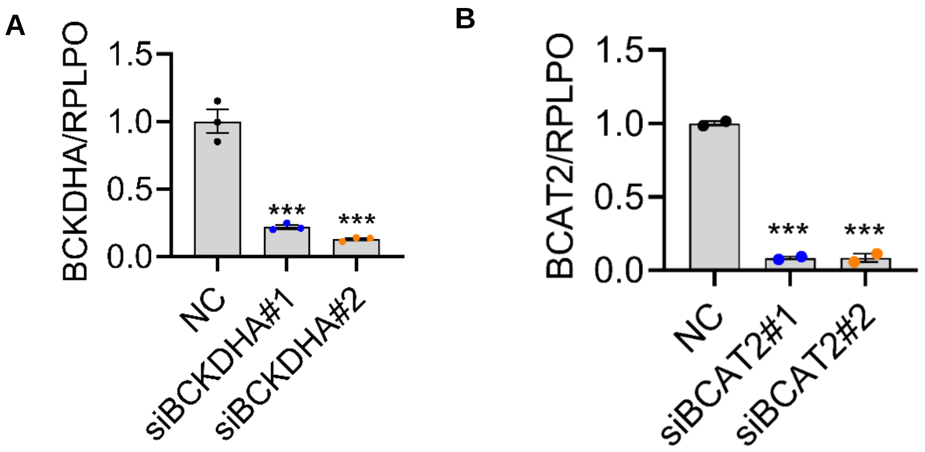

Figure S7. The expression of *BCKDHA* and *BCAT2* is reduced by siRNA-mediated knockdown in 769-P cells. 769-P cells were transiently transfected with 50 nM of either a negative control siRNA (NC) or siRNA targeting *BCKDHA* (A) or *BCAT2* (B) for 72 h. Data are presented as means ± SEM. Ordinary one-way ANOVA with Tukey's multiple comparison test was used. *** $P < 0.001$.

Table S1. KEGG pathway analysis representing that the pathways are negatively correlated with HDAC7 in TCGA data set.

RANK	GENE SET	P-VALUE
1	TCA cycle enzymes	1.60E-09
2	Parkinsons disease	4.80E-04
3	Oxidative phsphorylation	4.80E-04
4	Huntingtons disease	5.42E-04
5	Alzheimers disease	2.05E-03
6	BCAA catabolic enzymes	4.88E-03

Table S2. Summary of all significanlty differentially expressed metabolites in normal and tumor RCC samples

Shaded cells indicate p≤0.05 (red indicates that the mean values are significantly higher for that comparison; green values significantly lower). Blue-bolded text indicates 0.05<p<0.10.		Fold of Change	Statistical Values	
SUB PATHWAY	BIOCHEMICAL NAME	Tumor vs. Normal	Tumor vs. Normal	
			P-VALUE	Q-VALUE
Glycine, serine and threonine metabolism	glycine	0.93	0.4342	0.1158
	serine	0.56	< 0.001	0.0000
	N-acetylserine	0.62	0.0051	0.0026
	threonine	0.59	0.0034	0.0018
	N-acetylthreonine	0.79	0.0162	0.0071
	betaine	0.68	0.0072	0.0035
Alanine and aspartate metabolism	alanine	0.75	0.0361	0.0141
	beta-alanine	4.66	< 0.001	0.0000
	N-acetylalanine	1.03	0.7103	0.1778
	aspartate	0.41	< 0.001	0.0001
	N-acetylaspartate (NAA)	0.18	< 0.001	0.0000
	asparagine	0.60	0.0036	0.0019
	N-acetylasparagine	**1.63**	0.0835	0.0291
	3-ureidopropionate	0.77	0.1403	0.0453
Glutamate metabolism	glutamate	1.42	< 0.001	0.0004
	glutamate, gamma-methyl ester	3.24	< 0.001	0.0001
	glutamine	1.22	0.0063	0.0031
	pyroglutamine*	2.68	< 0.001	0.0000
	gamma-aminobutyrate (GABA)	0.36	< 0.001	0.0002
	socap	0.13	< 0.001	0.0000
	N-acetylglutamine	2.79	< 0.001	0.0000
Histidine metabolism	histidine	0.65	< 0.001	0.0002
	urocanate	0.57	< 0.001	0.0000
	1-methylimidazoleacetate	0.48	< 0.001	0.0000
Lysine metabolism	lysine	0.70	0.0046	0.0023
	2-aminoadipate	2.50	0.0146	0.0065
	pipecolate	1.28	0.0286	0.0116
	N2-acetyllysine	1.22	0.0052	0.0026
	N6-acetyllysine	0.30	< 0.001	0.0000
	glutaroyl carnitine	0.32	< 0.001	0.0000
Phenylalanine & tyrosine metabolism	phenylalanine	0.78	0.0129	0.0059
	p-cresol sulfate	0.77	0.4265	0.1152
	tyrosine	0.65	0.0010	0.0006
	3-(4-hydroxyphenyl)lactate	2.44	< 0.001	0.0000
	3-methoxytyrosine	1.05	0.5862	0.1504
	N-acetylphenylalanine	0.85	0.0102	0.0047
	phenylacetylglutamine	0.06	< 0.001	0.0000
	phenol sulfate	0.62	< 0.001	0.0003
Tryptophan metabolism	kynurenate	0.19	< 0.001	0.0000
	kynurenine	4.89	< 0.001	0.0000
	tryptophan	0.62	0.0014	0.0008
	indolelactate	1.92	< 0.001	0.0000
	tryptophan betaine	2.34	< 0.001	0.0000
	N-acetyltryptophan	**0.73**	0.0864	0.0300
	C-glycosyltryptophan*	**0.82**	0.0748	0.0267

Valine, leucine and isoleucine metabolism	3-methyl-2-oxobutyrate	**1.39**	0.0976	0.0332
	3-methyl-2-oxovalerate	1.60	0.2519	0.0743
	isoleucine	0.61	< 0.001	0.0001
	leucine	0.74	0.0067	0.0033
	N-acetylleucine	**1.22**	0.0580	0.0217
	valine	0.74	0.0082	0.0039
	4-methyl-2-oxopentanoate	1.52	0.8467	0.2065
	alpha-hydroxyisovalerate	1.64	< 0.001	0.0000
	isobutyrylcarnitine	0.43	< 0.001	0.0004
	2-methylbutyroylcarnitine	0.36	< 0.001	0.0000
	isovalerylcarnitine	0.32	< 0.001	0.0000
	hydroxyisovaleroyl carnitine	0.41	< 0.001	0.0000
	tiglyl carnitine	1.23	0.9052	0.2184
Cysteine, methionine, SAM, taurine metabolism	cysteine	1.43	0.5114	0.1339
	cystine	2.42	0.0167	0.0073
	methionine sulfoxide	0.55	< 0.001	0.0000
	hypotaurine	2.10	0.0075	0.0036
	taurine	1.20	0.3766	0.1038
	S-adenosylhomocysteine (SAH)	1.88	0.3364	0.0942
	methionine	0.62	< 0.001	0.0002
	N-acetylmethionine	3.28	< 0.001	0.0000
	2-hydroxybutyrate (AHB)	1.13	0.0199	0.0086
Urea cycle; arginine-, proline-, metabolism	dimethylarginine (SDMA + ADMA)	0.30	< 0.001	0.0000
	arginine	1.12	0.9585	0.2269
	N-acetylarginine	2.62	< 0.001	0.0000
	ornithine	0.55	< 0.001	0.0001
	urea	0.62	0.0230	0.0096
	proline	0.63	< 0.001	0.0001
	citrulline	0.70	0.0076	0.0036
	N-acetylornithine	0.99	0.7649	0.1897
	trans-4-hydroxyproline	1.11	0.4876	0.1292
	stachydrine	1.25	0.0217	0.0092
Creatine metabolism	creatine	1.66	< 0.001	0.0000
	creatinine	0.36	< 0.001	0.0000
Butanoate metabolism	2-aminobutyrate	1.35	0.0219	0.0092
Polyamine metabolism	5-methylthioadenosine (MTA)	0.64	< 0.001	0.0001
	spermidine	1.68	< 0.001	0.0001
	spermine	1.51	0.9400	0.2242
Guanidino and acetamido metabolism	4-guanidinobutanoate	2.12	0.0271	0.0111
	4-acetamidobutanoate	0.28	< 0.001	0.0000
Glutathione metabolism	glutathione, reduced (GSH)	**4.37**	0.0904	0.0311
	5-oxoproline	1.51	< 0.001	0.0001
	glutathione, oxidized (GSSG)	0.98	0.6406	0.1630
	cysteine-glutathione disulfide	1.10	0.2248	0.0677
	ophthalmate	2.33	0.5318	0.1381
Dipeptide	glycylvaline	0.84	0.0112	0.0051
	glycylproline	2.35	< 0.001	0.0001
	glycylleucine	1.44	0.2631	0.0771
	alanylleucine	0.68	0.1353	0.0440
	aspartylphenylalanine	2.84	0.0240	0.0100
	leucylleucine	0.82	0.1029	0.0346
Dipeptide derivative	anserine	0.35	< 0.001	0.0000
	gamma-glutamylleucine	1.86	< 0.001	0.0000

gamma-glutamyl	gamma-glutamylglutamate	3.88	< 0.001	0.0000
	gamma-glutamylglutamine	3.75	0.1058	0.0353
Polypeptide	VGAHAGEYGAEALER*	6.91	< 0.001	0.0000
Fibrinogen cleavage peptide	ADSGEGDFXAEGGGVR*	3.01	0.0045	0.0023
	ADpSGEGDFXAEGGGVR*	1.77	0.0456	0.0176
Aminosugars metabolism	N-acetylglucosamine 6-phosphate	1.75	< 0.001	0.0002
	erythronate*	0.71	0.0219	0.0092
	N-acetylneuraminate	3.31	< 0.001	0.0000
Fructose, mannose, galactose, starch, and sucrose metabolism	fructose	1.42	0.0094	0.0044
	maltose	5.84	< 0.001	0.0000
	mannitol	0.33	< 0.001	0.0005
	mannose	0.96	0.7188	0.1793
	mannose-6-phosphate	4.67	< 0.001	0.0000
	sorbitol	3.09	0.0012	0.0007
	sucrose	0.65	0.4269	0.1152
	maltotriose	10.21	< 0.001	0.0000
	raffinose	0.72	0.3812	0.1048
	maltotetraose	7.36	< 0.001	0.0000
	maltopentaose	4.67	< 0.001	0.0000
	maltohexaose	3.35	< 0.001	0.0000
Glycolysis, gluconeogenesis, pyruvate metabolism	1,5-anhydroglucitol (1,5-AG)	1.05	0.0248	0.0103
	glycerate	**0.79**	0.0907	0.0311
	glucose-6-phosphate (G6P)	6.48	< 0.001	0.0000
	glucose	1.72	< 0.001	0.0000
	fructose-6-phosphate	5.79	< 0.001	0.0000
	Isobar: fructose 1,6-diphosphate, glucose 1,6-diphosphate	2.71	0.0032	0.0017
	2-phosphoglycerate	**0.76**	0.0597	0.0222
	3-phosphoglycerate	0.69	0.5135	0.1341
	dihydroxyacetone phosphate (DHAP)	1.55	< 0.001	0.0006
	1,3-dihydroxyacetone	1.08	0.5685	0.1463
	phosphoenolpyruvate (PEP)	**0.53**	0.0556	0.0212
	lactate	1.26	0.1006	0.0341
	glucuronate	0.14	< 0.001	0.0000
Nucleotide sugars, pentose metabolism	6-phosphogluconate	1.94	0.0300	0.0121
	arabitol	0.43	< 0.001	0.0000
	ribitol	1.10	0.9860	0.2319
	threitol	0.32	< 0.001	0.0001
	erythrose-4-phosphate	0.72	0.5602	0.1446
	sedoheptulose-7-phosphate	2.13	0.0041	0.0021
	gluconate	0.31	0.4153	0.1127
	ribose	1.01	0.2520	0.0743
	ribose 5-phosphate	2.51	< 0.001	0.0001
	ribulose	**1.88**	0.0528	0.0202
	Isobar: ribulose 5-phosphate, xylulose 5-phosphate	2.66	< 0.001	0.0001
	xylitol	0.45	0.0019	0.0010
	xylulose	1.55	0.0094	0.0044
Krebs cycle	citrate	3.27	< 0.001	0.0001
	succinate	2.07	0.1768	0.0543
	succinylcarnitine	0.73	0.0040	0.0021
	fumarate	0.42	< 0.001	0.0000
	malate	0.45	0.0023	0.0013
	acetylphosphate	0.82	0.0108	0.0050

Oxidative phosphorylation	phosphate	0.73	0.0086	0.0041
	pyrophosphate (PPi)	0.62	< 0.001	0.0001
Essential fatty acid	linoleate (18:2n6)	1.26	0.0355	0.0139
	linolenate [alpha or gamma; (18:3n3 or 6)]	1.19	0.1303	0.0425
	dihomo-linolenate (20:3n3 or n6)	**1.87**	0.0592	0.0221
	eicosapentaenoate (EPA; 20:5n3)	0.73	0.1500	0.0477
	docosapentaenoate (n3 DPA; 22:5n3)	1.55	0.2474	0.0735
	docosapentaenoate (n6 DPA; 22:5n6)	**0.94**	0.0731	0.0262
	docosahexaenoate (DHA; 22:6n3)	0.48	< 0.001	0.0001
Medium chain fatty acid	caproate (6:0)	0.40	< 0.001	0.0000
	caprylate (8:0)	1.24	0.1139	0.0377
	pelargonate (9:0)	1.07	0.1670	0.0520
	laurate (12:0)	1.05	0.4908	0.1297
Long chain fatty acid	myristate (14:0)	0.98	0.6731	0.1707
	pentadecanoate (15:0)	0.96	0.8495	0.2066
	palmitate (16:0)	**1.14**	0.0564	0.0214
	palmitoleate (16:1n7)	**1.12**	0.0723	0.0260
	margarate (17:0)	1.19	0.1275	0.0418
	10-heptadecenoate (17:1n7)	1.24	0.0289	0.0117
	stearate (18:0)	1.25	0.0012	0.0007
	oleate (18:1n9)	**1.64**	0.0568	0.0215
	cis-vaccenate (18:1n7)	1.52	0.0340	0.0135
	stearidonate (18:4n3)	1.31	0.1514	0.0478
	nonadecanoate (19:0)	2.16	< 0.001	0.0000
	10-nonadecenoate (19:1n9)	2.49	< 0.001	0.0000
	arachidate (20:0)	4.41	< 0.001	0.0000
	eicosenoate (20:1n9 or 11)	4.73	< 0.001	0.0000
	dihomo-linoleate (20:2n6)	3.28	< 0.001	0.0000
	arachidonate (20:4n6)	0.48	< 0.001	0.0002
	docosadienoate (22:2n6)	4.51	< 0.001	0.0000
	docosatrienoate (22:3n3)	4.56	< 0.001	0.0000
	adrenate (22:4n6)	1.98	< 0.001	0.0004
Fatty acid, monohydroxy	4-hydroxybutyrate (GHB)	1.44	0.6154	0.1570
	2-hydroxystearate	0.42	< 0.001	0.0006
	2-hydroxypalmitate	0.27	< 0.001	0.0000
	3-hydroxysebacate	0.45	< 0.001	0.0000
	13-HODE + 9-HODE	0.21	< 0.001	0.0000
Fatty acid, dicarboxylate	adipate	**0.80**	0.0672	0.0245
	2-hydroxyglutarate	5.36	< 0.001	0.0005
	azelate (nonanedioate)	0.44	< 0.001	0.0000
	tetradecanedioate	0.87	0.7685	0.1901
	hexadecanedioate	0.64	0.1241	0.0408
	3-carboxy-4-methyl-5-propyl-2-furanpropanoate (CMPF)	1.09	0.0332	0.0132
Eicosanoid	prostaglandin E2	0.35	< 0.001	0.0001
	6-keto prostaglandin F1alpha	**0.75**	0.0713	0.0257
	5-HETE	0.10	< 0.001	0.0000
	15-HETE	0.13	< 0.001	0.0000
Fatty acid metabolism (also BCAA metabolism)	propionylcarnitine	0.73	0.0211	0.0090
	butyrylcarnitine	1.91	< 0.001	0.0000
Fatty acid metabolism	valerylcarnitine	0.49	0.0013	0.0007
Fatty acid synthesis	malonylcarnitine	0.34	< 0.001	0.0000
	deoxycarnitine	1.45	0.0025	0.0014

Carnitine metabolism	carnitine	1.39	0.0136	0.0061
	3-dehydrocarnitine*	1.08	0.9142	0.2198
	acetylcarnitine	2.03	< 0.001	0.0000
	hexanoylcarnitine	1.89	< 0.001	0.0000
	octanoylcarnitine	2.61	< 0.001	0.0000
	laurylcarnitine	1.76	< 0.001	0.0000
	palmitoylcarnitine	1.45	0.1473	0.0470
	stearoylcarnitine	1.00	0.4992	0.1315
	oleoylcarnitine	**1.57**	0.0754	0.0268
Bile acid metabolism	taurocholate	0.67	< 0.001	0.0005
	glycochenodeoxycholate	1.48	0.0050	0.0025
	glycolithocholate sulfate*	0.83	0.3193	0.0903
	taurolithocholate 3-sulfate	0.96	0.1709	0.0529
	glycocholenate sulfate*	0.96	0.7111	0.1778
	taurocholenate sulfate*	**0.98**	0.0573	0.0215
Glycerolipid metabolism	choline phosphate	1.15	0.4341	0.1158
	ethanolamine	0.33	< 0.001	0.0000
	phosphoethanolamine	0.90	0.1842	0.0562
	glycerophosphoethanolamine	0.50	< 0.001	0.0000
	glycerol	1.19	0.6921	0.1746
	choline	**0.88**	0.0619	0.0228
	glycerol 3-phosphate (G3P)	0.62	0.7422	0.1846
	glycerophosphorylcholine (GPC)	1.63	0.0147	0.0065
	cytidine 5'-diphosphocholine	1.57	0.9750	0.2302
Inositol metabolism	myo-inositol	0.39	< 0.001	0.0000
	chiro-inositol	0.38	0.1180	0.0390
	inositol 1-phosphate (I1P)	0.57	0.0013	0.0007
	scyllo-inositol	0.20	< 0.001	0.0000
Ketone bodies	3-hydroxybutyrate (BHBA)	1.06	0.1030	0.0346
	1,2-propanediol	0.84	0.8082	0.1977
Lysolipid	1-palmitoylglycerophosphoethanolamine	0.03	< 0.001	0.0000
	2-palmitoylglycerophosphoethanolamine*	0.61	0.0301	0.0121
	1-stearoylglycerophosphoethanolamine	0.10	< 0.001	0.0000
	1-oleoylglycerophosphoethanolamine	0.09	< 0.001	0.0000
	2-oleoylglycerophosphoethanolamine*	0.33	< 0.001	0.0000
	1-linoleoylglycerophosphoethanolamine*	0.27	< 0.001	0.0000
	2-linoleoylglycerophosphoethanolamine*	0.61	0.0325	0.0130
	1-arachidonoylglycerophosphoethanolamine*	0.64	< 0.001	0.0001
	2-arachidonoylglycerophosphoethanolamine*	0.69	0.1470	0.0470
	2-docosapentaenoylglycerophosphoethanolamine*	0.96	0.1718	0.0530
	2-docosahexaenoylglycerophosphoethanolamine*	0.73	0.1842	0.0562
	1-myristoylglycerophosphocholine	0.29	< 0.001	0.0000
	1-palmitoylglycerophosphocholine	0.25	< 0.001	0.0006
	2-palmitoylglycerophosphocholine*	1.23	0.9161	0.2198
	1-palmitoleoylglycerophosphocholine*	0.25	< 0.001	0.0000
	2-palmitoleoylglycerophosphocholine*	2.38	< 0.001	0.0004
	1-stearoylglycerophosphocholine	0.29	< 0.001	0.0005
	1-oleoylglycerophosphocholine	0.28	< 0.001	0.0004
	2-oleoylglycerophosphocholine*	**0.54**	0.0792	0.0279
	1-linoleoylglycerophosphocholine	0.49	0.0269	0.0111
	2-linoleoylglycerophosphocholine*	**0.63**	0.0869	0.0300
	2-arachidonoylglycerophosphocholine*	0.89	0.5114	0.1339
	1-palmitoylglycerophosphoinositol*	0.17	< 0.001	0.0000

	1-stearoylglycerophosphoinositol	0.23	< 0.001	0.0000
	1-arachidonoylglycerophosphoinositol*	0.86	0.5416	0.1402
	2-arachidonoylglycerophosphoinositol*	1.23	0.9396	0.2242
	1-oleoylglycerophosphoserine	**0.73**	0.0644	0.0236
	2-oleoylglycerophosphoserine*	0.97	0.2715	0.0793
Monoacylglycerol	2-palmitoylglycerol (2-monopalmitin)	0.32	0.0011	0.0006
	1-oleoylglycerol (1-monoolein)	1.08	0.6069	0.1553
Sphingolipid	sphinganine	1.05	0.4315	0.1158
	sphingosine	1.01	0.7741	0.1909
	phytosphingosine	0.65	0.0036	0.0019
	palmitoyl sphingomyelin	0.82	0.0400	0.0155
Sterol/Steroid	squalene	0.55	< 0.001	0.0000
	cholesterol	1.07	0.9421	0.2242
	dihydrocholesterol	2.22	0.2431	0.0724
	7-alpha-hydroxycholesterol	0.33	< 0.001	0.0000
	7-beta-hydroxycholesterol	0.25	< 0.001	0.0000
	dehydroisoandrosterone sulfate (DHEA-S)	0.60	0.0016	0.0009
	epiandrosterone sulfate	0.58	0.0137	0.0061
	androsterone sulfate	0.74	0.2186	0.0660
	cortisol	1.46	< 0.001	0.0000
	cortisone	0.26	< 0.001	0.0000
	7-alpha-hydroxy-3-oxo-4-cholestenoate (7-Hoca)	3.55	< 0.001	0.0000
	4-androsten-3beta,17beta-diol disulfate 1*	0.95	0.1518	0.0478
	5alpha-androstan-3beta,17beta-diol disulfate	0.87	0.1597	0.0499
	pregnen-diol disulfate*	0.82	0.1699	0.0527
	andro steroid monosulfate 1*	0.73	0.0059	0.0029
	andro steroid monosulfate 2*	0.56	< 0.001	0.0000
Purine metabolism, (hypo)xanthine/inosine containing	xanthine	0.85	0.1515	0.0478
	xanthosine	0.61	< 0.001	0.0001
	hypoxanthine	0.85	0.1392	0.0451
	inosine	1.71	0.0186	0.0081
	2'-deoxyinosine	1.41	0.5315	0.1381
Purine metabolism, adenine containing	adenine	0.74	0.0130	0.0059
	adenosine	0.39	< 0.001	0.0000
	N1-methyladenosine	0.38	< 0.001	0.0000
	adenosine 2'-monophosphate (2'-AMP)	1.03	0.2316	0.0695
	adenosine 3'-monophosphate (3'-AMP)	0.29	< 0.001	0.0000
	adenosine 5'-monophosphate (AMP)	1.49	0.0015	0.0008
Purine metabolism, guanine containing	guanosine	1.25	0.6810	0.1723
	2'-deoxyguanosine	1.60	0.4084	0.1112
	guanosine 5'- monophosphate (GMP)	2.40	0.0253	0.0105
	N1-methylguanosine	0.30	< 0.001	0.0000
	N2-methylguanosine	0.41	< 0.001	0.0000
	N2,N2-dimethylguanosine	0.25	< 0.001	0.0000
	N6-carbamoylthreonyladenosine	0.33	< 0.001	0.0000
Purine metabolism, urate metabolism	urate	0.93	0.1423	0.0458
Pyrimidine metabolism, cytidine containing	cytidine	1.19	0.0211	0.0090
	cytidine 5'-monophosphate (5'-CMP)	1.97	< 0.001	0.0000
	cytidine-3'-monophosphate (3'-CMP)	0.72	0.0069	0.0034
Pyrimidine metabolism, thymine containing	thymine	1.20	0.3409	0.0952
Pyrimidine metabolism, thymine containing;	3-aminoisobutyrate	2.40	< 0.001	0.0000
Pyrimidine metabolism, [illegible]	uracil	0.72	0.1043	0.0349
	uridine	1.09	0.2017	0.0611

Pyrimidine metabolism, uracil containing	pseudouridine	0.52	< 0.001	0.0000
	5-methyluridine (ribothymidine)	0.76	0.0055	0.0027
	uridine 5'-monophosphate (UMP)	2.64	0.1940	0.0590
Purine and pyrimidine metabolism	methylphosphate	0.77	0.0101	0.0047
Ascorbate and aldarate metabolism	ascorbate (Vitamin C)	2.25	0.3686	0.1026
	dehydroascorbate	1.53	0.7953	0.1954
	threonate	1.32	0.9874	0.2319
	arabonate	1.06	0.8854	0.2142
Folate metabolism	5-methyltetrahydrofolate (5MeTHF)	0.25	< 0.001	0.0000
Hemoglobin and porphyrin	heme*	1.54	0.3865	0.1056
Hemoglobin and porphyrin metabolism	bilirubin (Z,Z)	1.26	0.0365	0.0142
	bilirubin (E,E)*	1.07	0.1544	0.0484
	biliverdin	1.66	< 0.001	0.0000
Nicotinate and nicotinamide metabolism	nicotinamide	0.95	0.3863	0.1056
	nicotinamide ribonucleotide (NMN)	**1.19**	0.0808	0.0284
	nicotinamide adenine dinucleotide (NAD+)	0.44	< 0.001	0.0000
	nicotinamide riboside*	6.96	< 0.001	0.0002
	adenosine 5'diphosphoribose	1.36	0.3722	0.1030
	nicotinate ribonucleoside*	**1.22**	0.0766	0.0271
	1-methylnicotinamide	3.14	< 0.001	0.0000
	trigonelline (N'-methylnicotinate)	0.32	< 0.001	0.0000
Pantothenate and CoA metabolism	pantothenate	0.33	< 0.001	0.0000
	phosphopantetheine	0.36	< 0.001	0.0000
	3'-dephosphocoenzyme A	0.40	< 0.001	0.0000
	acetyl CoA	0.80	0.0106	0.0049
Riboflavin metabolism	flavin adenine dinucleotide (FAD)	0.27	< 0.001	0.0000
	riboflavin (Vitamin B2)	0.33	< 0.001	0.0000
Tocopherol metabolism	alpha-tocopherol	7.01	< 0.001	0.0000
	gamma-tocopherol	2.93	< 0.001	0.0000
Vitamin B6 metabolism	pyridoxate	0.11	< 0.001	0.0000
Benzoate metabolism	hippurate	0.09	< 0.001	0.0000
	2-hydroxyhippurate (salicylurate)	0.34	< 0.001	0.0001
	4-hydroxyhippurate	0.04	< 0.001	0.0000
	catechol sulfate	0.69	0.0140	0.0062
Chemical	glycolate (hydroxyacetate)	1.01	0.9584	0.2269
	glycerol 2-phosphate	1.40	0.7966	0.1954
	triethyleneglycol	0.71	0.8661	0.2101
	heptaethylene glycol	0.51	0.0457	0.0176
	hexaethylene glycol	**0.61**	0.0600	0.0222
	tetraethylene glycol	0.57	0.0342	0.0135
	methyl-alpha-glucopyranoside	2.52	< 0.001	0.0000
Drug	ofloxacin	0.99	0.2390	0.0714
	4-acetaminophen sulfate	0.48	0.0035	0.0019
	4-acetamidophenol	0.97	0.3215	0.0903
	p-acetamidophenylglucuronide	0.28	0.0016	0.0009
	2-hydroxyacetaminophen sulfate*	0.51	< 0.001	0.0000
	2-methoxyacetaminophen sulfate*	0.92	0.1084	0.0360
	3-(cystein-S-yl)acetaminophen*	0.30	0.0065	0.0032
	ibuprofen	1.41	0.3215	0.0903
	naproxen	1.01	0.3215	0.0903
	desmethylnaproxen sulfate*	0.99	0.3215	0.0903
	lidocaine	0.89	0.3708	0.1029
	ketamine	1.04	0.3215	0.0903

	metoprolol	**0.94**	0.0663	0.0242
	metoprolol acid metabolite*	**0.64**	0.0705	0.0255
	N-ethylglycinexylidide*	**0.76**	0.0817	0.0286
	carbamazepine*	1.00	0.3215	0.0903
	carbamazepine 10,11-epoxide*	0.99	0.3215	0.0903
	carbamazepine glucuronide*	0.84	0.3215	0.0903
	gabapentin	**0.50**	0.0947	0.0323
	meprobamate*	1.00	0.3215	0.0903
	atenolol	1.00	0.4792	0.1274
	citalopram	0.99	0.3215	0.0903
	ranitidine	0.94	0.3215	0.0903
Food component/Plant	ergothioneine	3.50	< 0.001	0.0000
Xanthine metabolism	caffeine	1.28	0.0028	0.0015
	paraxanthine	1.01	0.6987	0.1757
	theobromine	1.06	0.4335	0.1158
	1,7-dimethylurate	0.35	< 0.001	0.0000
Sugar, sugar substitute, starch	erythritol	0.87	0.2544	0.0748

Table S3. Genes associated with BCAA catabolism and glycolysis used in in Volcano plot

BCAA genes	Glycolysis genes
BCAT1	HK2
BCAT2	GPI
DLD	PFKL
AUH	ALDOA
ACAD8	ALDOC
ALDH3A2	TPI1
ACAT2	GAPDH
HADHB	PGK1
MCCC2	PGAM1
PCCB	PGAM2
HSD17810	ENO1
HADHA	ENO2
HIBADH	PKLR
ALDH9A1	
MCCC1	
MCEE	
BCKDHA	
DBT	
HIBCH	
MUT	
PCCA	
HADH	
BCKDHB	
ALDH1B1	
ACADM	
IVD	
HMGCL	
ECHS1	
HMGCS2	
ACAT2	
EHHADH	
ABAT	
ACAA1	
ACADSB	
ACAA2	
ALDH2	
ACADSB	
ALDH6A1	

Table S4. Pre-designed Taqman primers used for RT-qPCR

NAME	ASSAY ID
BCKDHA	Hs00958109_m1
BCKDHB	Hs00609053_m1
ACADM	Hs00936584_m1
ACADSB	Hs00155631_m1
HADHB	Hs01027272_m1
MUT	Hs01071471_m1
DLD	Hs00164401_m1
DBT	Hs01066445_m1
ALDH6A1	Hs00194421_m1
AUH	Hs01060463_m1
BCAT2	Hs01553550_m1
HDAC7	Hs00248789_m1
HIBCH	Hs00961835_g1
MCC1	Hs00894983_m1
SNAI1	Hs00195591_m1
RPLPO	Hs 99999902_ m1

Table S5. Primers used for ChIP-qPCR

NAME	PRIMER SEQUENCES
DLD-pro-F	5'-GGTTGTTGCAAGGATGAAATGA-3'
DLD-pro-R	5'-TATCGACAGTAAAGCGTGACAA-3'
AUH-Pro-F	5'-CACGCCGTAAACAGACAACA-3'
AUH-Pro-R	5'-GAGCCACGCACTGCAAG-3'
ACADM-Pro-F	5'-CAGAGGTGGAAACGCAGAAA-3'
ACADM-Pro-R	5'-CATGCTCCGTGACCCTTG-3'
BCKDHA-Pro-F1	5'-GGCTTAGCGTTTACCAACAATC-3'
BCKDHA-Pro-R1	5'-CCCAGAGTAAAGCGGATCAAT-3'
BCKDHA-Pro-F2	5'-TCAGGTTGCCCTCTTCTTTG-3'
BCKDHA-Pro-R2	5'-TCTTCCCGTTCTGTGGTAGATA -3'

Table S6. List of antibodies used in this study

NAME	SPECIES	REFERENCE NUMBER
HDAC7	Mouse	Sc-74563x (Santa Cruz)
HADHB	Mouse	Sc-271495 (Santa Cruz)
DLD	Rabbit	NBP1-31302 (Novus Biologicals)
AUH	Rabbit	NBP2-92807 (Novus Biologicals)
HIBADH	Mouse	Sc-398288 (Santa Cruz)
ACADSB	Mouse	Sc-398773 (Santa Cruz)
BCKDHA	Rabbit	NBP1-79616 (Novus Biologicals)
BCKDHA	Mouse	Sc-271538 (Santa Cruz)
BCKDHB	Mouse	Sc-374630 (Santa Cruz)
BCAT2	Rabbit	16417-1-AP (Proteintech)
PPARGC1A	Rabbit	NBP1-04676S (Novus Biological)
ACADS	Rabbit	HPA022271 (Sigma-Aldrich)
SNAI1	Rabbit	3879S (CST)
NICD	Rabbit	#4147 (CST)
HES1	Rabbit	#11988 (CST)
Tubulin	Mouse	T5168 (Sigma-Aldrich)
H3K27Ac	Rabbit	#4535 (CST)
Rabbit IgG	Rabbit	12-370 (Millipore)
